# Endosomal sorting results in a selective separation of the protein corona from nanoparticles

**DOI:** 10.1038/s41467-023-35902-9

**Published:** 2023-01-18

**Authors:** Shen Han, Richard da Costa Marques, Johanna Simon, Anke Kaltbeitzel, Kaloian Koynov, Katharina Landfester, Volker Mailänder, Ingo Lieberwirth

**Affiliations:** 1 Max Planck Institute for Polymer Research, Ackermannweg 10, 55128 Mainz, Germany; 2 Dermatology Clinic, University Medical Center of the Johannes Gutenberg-University Mainz, Langenbeckstr. 1, 55131 Mainz, Germany

**Keywords:** Drug delivery, Biopolymers

## Abstract

The formation of the protein corona is a well-known effect when nanoparticles (NP) are exposed to biological environments. The protein corona is the most important factor, which determines the rate and route of endocytosis, and decisively impacts cellular processes and even the release of the active pharmaceutical ingredient from the nanoparticles. While many studies concentrate on the effect of the protein corona formation extracellularly or the uptake consequences, little is known about the fate of the protein corona inside of cells. Here, we reconstruct for the first time the separation of the protein corona from the NPs by the cell and their further fate. Ultimately, the NPs and protein corona are separated from each other and end up in morphologically different cellular compartments. The cell directs the NPs towards recycling endosomes, whereas the protein corona gathers in multivesicular bodies. From this, we conclude that the NPs are prepared for subsequent exocytosis, while the protein corona remains in the cell and is finally metabolized there.

## Abstract (web summary)

Protein corona formation on nanoparticles and the resultant effects on cellular interactions is well documented, where less is known about the fate of the corona in the cell. Here, the authors track the protein corona and nanoparticles in cells and describe the separation and different processing within different cellular compartments.

## Introduction

While classical drug development for small molecules tweaks the pharmacokinetic by changing the drug itself, nanomedicine is a fundamentally different approach in which active pharmaceutically ingredients (API) are incorporated into smart, nanoparticulate systems in order to achieve this. For example, the RNA-based Covid-19 vaccine from BioNTech/Pfizer is based precisely on this nanotechnology and shows the tremendous success of this concept^1,2^. The problem of stability of the mRNA outside of cells, as well as the uptake of a large mRNA molecule, is solved by a clever design of nanomedical packaging. After the application to the organism, the nanoparticles (NPs) will finally enter the targeted cells to release their API and achieve the desired pharmaceutical effect. Before that, however, the NPs need to be introduced into the body and hence come immediately into contact with a biological environment, which contains proteins and other biomolecules. As a result, a coat of biomolecules adsorbs instantaneously on the NPs termed protein corona^3^. This protein corona alters the NP surface from a chemical into a biological identity, which can impact blood circulation^4^, cellular uptake^5,6^, cytotoxicity^7^, and the release of the API from the NP^8,9^. Consequently, researchers aimed to form the protein corona in a controlled way to achieve tailored effects. These artificial protein coronas have been shown to actively induce cell type^10^ and tissue targeting^11^ or even prevent cellular uptake^12^, demonstrating the possibilities of pre-adsorbed proteins for nanoparticle modification.

However, it is yet unknown what happens to the protein corona after a NP is taken up by a cell, although detailed knowledge of the intracellular fate of the NP-associated protein corona is crucial for the understanding and development of any drug delivery system. Previous studies have highlighted the dynamic nature of the protein corona and the continuous exchange of the adsorbed proteins after the transition of the NPs to different protein-containing media. These studies demonstrate the exchange of proteins in both, single protein solutions and complex protein mixtures^13,14^. The protein exchange of the protein corona occurs also within the confined subcellular environment after internalization of the NPs with a distinct protein corona composition^15^. Eventually, Bertoli et. al. found evidence for the intracellular degradation of the protein corona from the NP in lysosomes using optical microscopy methods^16^.

These studies rely on staining distinct subcellular compartments but lack the ultrastructural aspects as detailed in electron microscopy. Here, we use a fluorescence staining technique combined with electron microscopy, termed correlative light and electron microscopy (CLEM). This allows us to obtain unbiased information about the subcellular fate of NPs and the protein corona. Moreover, even the unlabeled cellular environment of the protein corona becomes visible due to the unspecific imaging capacities supplied by the electron microscopy technique.

Here, we demonstrate the co-internalization and the subsequent in-cell separation of the protein corona from the NP in murine macrophages by combining confocal laser scanning microscopy (cLSM) with electron microscopy (EM). To realize a trackable protein corona, we fluorescently labeled murine plasma proteins and formed a fluorescent protein corona on NPs. For the first time, we performed single-cell volume CLEM to visualize the protein corona and the NPs in 3D within the cell with high resolution and reveal their subcellular location and fate by fluorescence. After co-internalization of the fluorescently labeled NPs together with the likewise fluorescently labeled protein corona, we observe a distinct decrease of the fluorescence signals over time and a spatial separation of the NPs from the protein corona signal in cLSM. To increase the resolution and also obtain information about the non-labeled cellular environment, we additionally performed EM studies and correlated them for the very same cells with the cLSM data. In particular, we have been able to reconstruct the volume of complete cells using CLEM array tomography, utilizing fluorescence data to localize the protein corona and NPs. These volume reconstructions clearly show an enrichment of the NPs and the protein corona in morphologically different compartments 24 h after incubation: The NPs are found in recycling endosomes (REs) whereas the protein corona is preferably found in multivesicular bodies (MVBs). Moreover, we found evidence for a subsequent exocytosis of the separated NPs and protein corona. Hence, these observations might impact the further design of drug delivery systems.

## Results and discussion

### The formation of a fluorescent protein corona

**Fig. 1:**
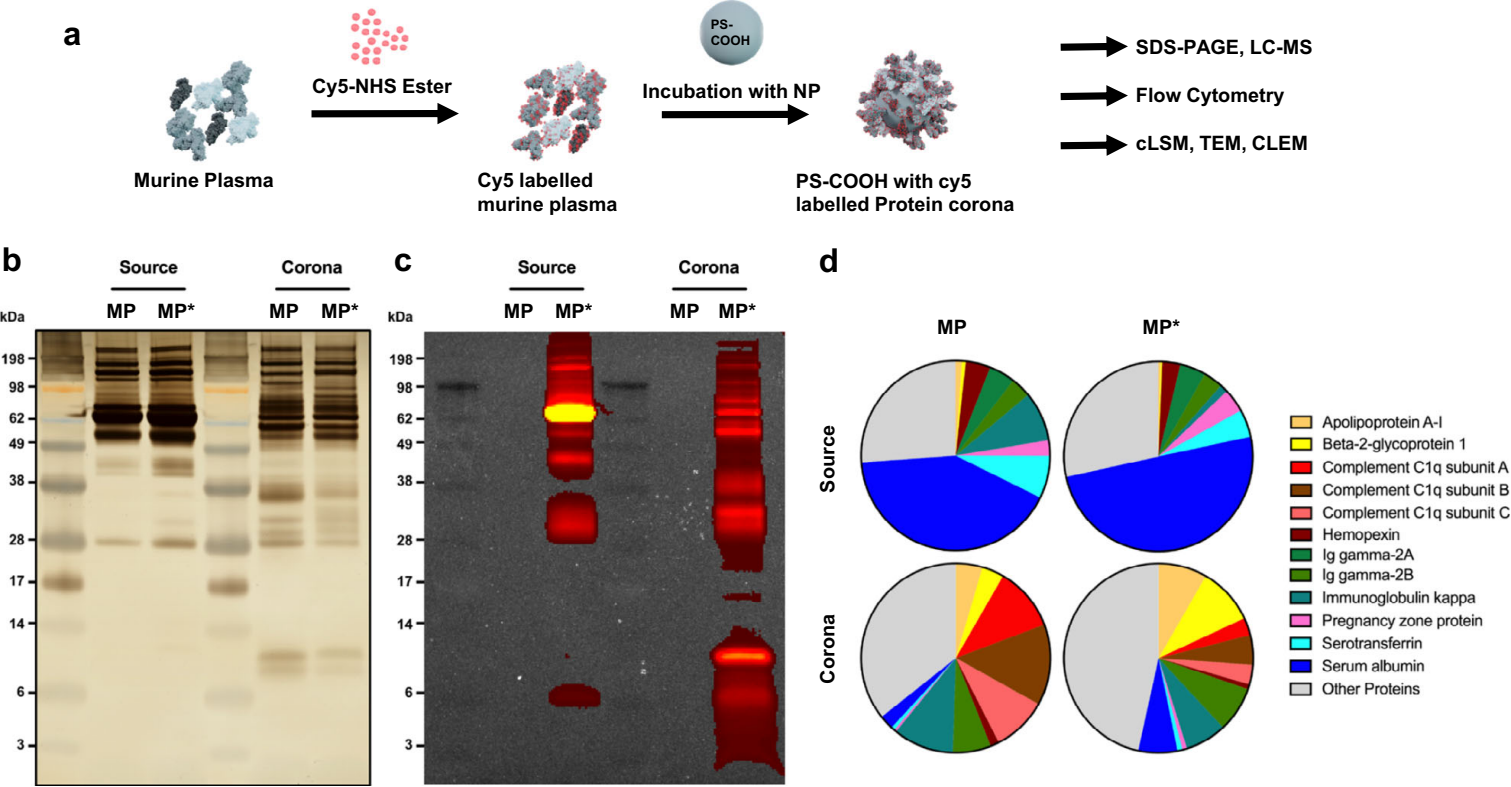
Protein analysis of unlabeled and Cy5-labeled murine plasma and protein corona. **a** Murine plasma proteins were labeled with Cy5 by NHS-chemistry. Carboxyl-functionalized PS NPs were incubated in unlabeled and Cy5-labeled murine plasma, respectively, to form a protein corona. **b** Unlabeled murine plasma (MP), Cy5-labeled murine plasma (MP*), and associated protein corona samples were analyzed by SDS-PAGE and silver staining. Corona proteins were obtained after incubation of carboxyl-functionalized PS NPs in plasma, washing, and desorption with 2% of SDS. **c** Analysis of in-gel fluorescence. The Cy5-fluorescence was imaged by IVIS at an excitation wavelength of 640 nm and an emission wavelength of 680 nm. **d** Quantitative LC-MS proteomic analysis. The pie charts display the proteins with at least 4% presence in the proteome. Values are represented as the percentage based on all identified proteins.

In order to form the protein corona and study its fate after cellular internalization, we synthesized and carboxyl-functionalized polystyrene nanoparticles (PS NPs). The synthesized PS NPs were stabilized with Lutensol, fluorescently labeled with BODIPY, and showed an average diameter of 116 nm and a zeta potential of 7.21 mV (Supplementary Table 1). We labeled the murine plasma proteins with Cy5 by NHS-chemistry and formed the protein corona on the NPs with both, unlabeled and labeled proteins (Fig. 1a).

Subsequently, we quantified the proteins and characterized the composition in the unlabeled and labeled plasma and unlabeled and labeled protein corona. The protein corona samples showed a comparable protein amount after desorption. We measured 51.79 (±1.14) µg for the unlabeled protein corona and 60.47 (±2.8) µg for the Cy5-labeled corona (proteins amounts as mean ± SD, *n* = 2, amounts correspond to 0.01 m^2^ NP surface). Additionally, we observed similar band patterns on the SDS-PAGE by silver staining between unlabeled and labeled samples, respectively (Fig. 1b, Supplementary Fig. 1). We detected the fluorescent labeling via in-gel fluorescence measurements by IVIS. Here we observed a high similarity between the band patterns of the silver-stained SDS Page and the in-gel fluorescence (Fig. 1c). This similarity confirmed the high degree of fluorescent labeling of the proteins of different molecular weights.

Next, we employed quantitative LC-MS measurements to further detail the protein composition. Overall, the protein composition was slightly changed in quantitative measures after the Cy5-labeling. Serum albumin was measured as the highest abundant protein in both, unlabeled and labeled murine plasma, followed by immunoglobulin kappa in the unlabeled plasma and serotransferrin in the labeled plasma as the second-highest proteins, respectively. For the unlabeled protein corona, we measured complement C1q subunit B and complement C1q subunit A as the two most abundant proteins. In the labeled protein corona we identified beta-2-glycoprotein and apolipoprotein A1 as the two most abundant proteins. After the Cy5-labeling, we observed changes in the protein composition of the murine plasma, such as a ~6 fold decrease of immunoglobulin kappa and a ~2.7 fold decrease of apolipoprotein A1. In the case of the protein corona samples, we determined a ~2.7 fold to ~3.6 fold decrease of the complement C1q subunits and a ~2.7 fold increase of serum albumin after labeling (Fig. 1d). For the remaining most abundant proteins, however, we observed smaller quantitative differences, as the ones mentioned. As a consequence, we accepted the described quantitative changes after the labeling and assumed that the labeling would not alter the outcome of a fluorescently labeled protein corona compared to an untreated protein corona. Nevertheless, we included the NPs with an unlabeled protein corona as a control in the following uptake experiments.

### Co-internalization of PS NPs and the protein corona

**Fig. 2:**
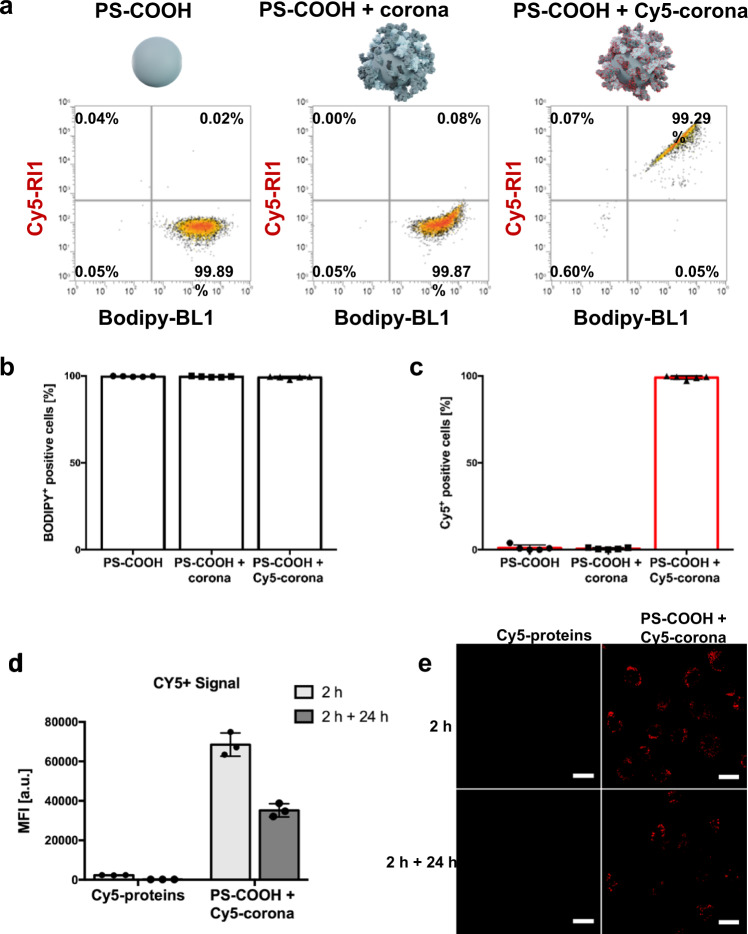
Cy5-labeled protein corona is detectable after uptake in murine macrophages. **a** RAW264.7 cells were incubated with 150 µg mL^−1^ of carboxyl-functionalized PS NPs for 2 h. Untreated NPs, NPs with an unlabeled protein corona, and NPs with a Cy5-labeled protein corona were used for the uptake experiment. Flow cytometry was performed to measure BODIPY (PS NPs) fluorescence and Cy5 (labeled corona proteins) fluorescence. Values are shown as the percentage of measured events regarding the fluorescence. **b** The percentage of BODIPY-positive cells is shown **c** The percentage of Cy5-positive cells is shown (data is shown as mean ± SD, *n* = 5). **d** As a control experiment, RAW264.7 cells were incubated with 9 µg mL^−1^ Cy5-labeled murine plasma proteins and 150 µg mL^−1^ of PS NPs with a Cy5-labeled protein corona, respectively. The amount of 9 µg mL^−1^ corresponds to the amount of proteins on the protein corona. The uptake was evaluated after 2 h and 2 h + 24 h. Flow cytometry was performed to measure the median fluorescence intensity (MFI) of Cy5 (proteins or protein corona; data is shown as mean ± SD, *n* = 3). The gating strategy for flow cytometry is provided in Supplementary Fig. 2. **e** The uptake was analyzed by cLSM at three different areas on a single section, and similar results were obtained. Red represents the Cy5-labeled protein corona. The green signal is not included for a better overview of the red signal. Scale bars: 20 µm.

Next, we employed flow cytometry to understand the uptake of both, PS NPs and the protein corona. Murine macrophages RAW264.7 were incubated with untreated PS NPs, PS NPs with an unlabeled protein corona, and PS NPs with a Cy5-labeled protein corona (Fig. 2a). After an incubation of 2 h with the nanoparticles, almost 100% of the cells were BODIPY-positive, indicating the uptake of the PS NPs by most cells (Fig. 2b). Almost all measured cells were Cy5-positive, if the cells were incubated with PS NPs with a Cy5-labeled protein corona (Fig. 2c). A control uptake experiment with only Cy5-labeled proteins showed that no or a fairly small amount of Cy5 proteins were taken up when compared to the uptake of Cy5-labeled proteins on the protein corona on PS NPs. This difference was confirmed by flow cytometry and cLSM (Fig. 2d/e). Therefore, our findings prove a co-internalization of the PS NPs and the associated protein corona.

### Visualizing PS NPs and protein corona within the cells

**Fig. 3:**
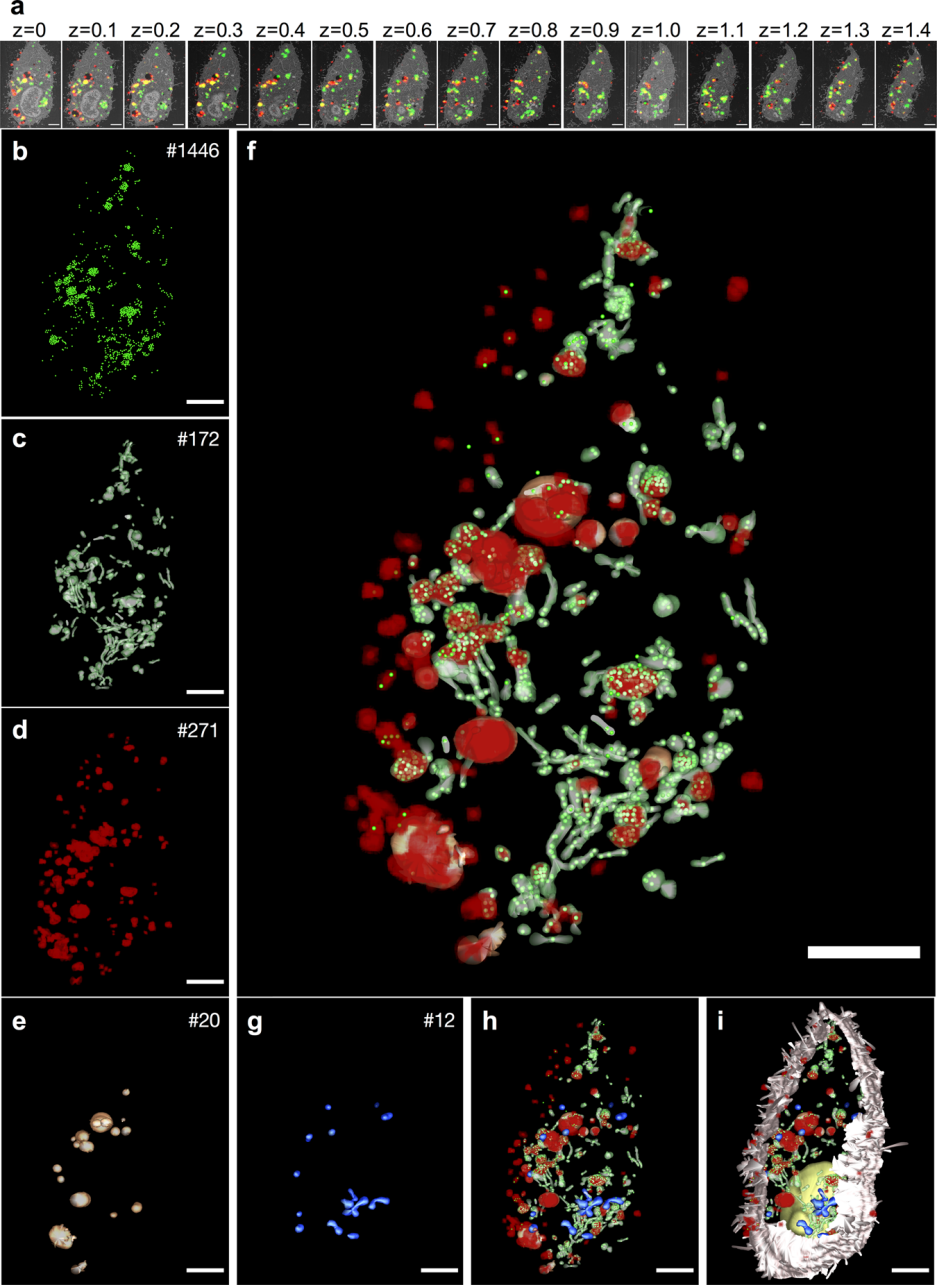
Volume CLEM of one cell and segmented model. PS NPs with Cy5-labeled protein corona were incubated with RAW264.7 cells and imaged in CLEM after 2 h + 24 h. **a** CLEM micrographs of 15 physical EPON sections (100 nm each). z represents the relative depth of each section in µm. Red represents the Cy5-labeled protein corona, green represents BODIPY-labeled PS NPs, yellow represents the overlay of the protein corona and PS NPs. The enlarged images are provided in Supplementary Fig. 3. **b–i** Segmented model of individual or combined objects with the number of segmented objects (upper right corner). **b** PS NPs **c** Vesicles containing PS NPs. **d** Protein corona **e** Vesicles containing protein corona **f** Combined objects of the protein corona, PS NPs, vesicles containing PS NPs, vesicles containing protein corona **g**. Mitochondria **h** Objects in **f** with mitochondria **i** objects in h with cell membrane (light pink) and nucleus (yellow). Scale bars: 2 µm.

In order to study the intracellular behavior of the protein corona, we performed volume CLEM in RAW264.7 macrophages with a Cy5-labeled protein corona on BODIPY-labeled PS NPs. Volume CLEM is an imaging technique that is applied to visualize the three-dimensional information of biological samples (e.g. cells, tissues) by combining the strength of light microscopy and electron microscopy. The imaging process is performed on serial sections. After 24 h of co-incubation of protein corona-coated PS NPs with macrophages, samples were immediately vitrified by high pressure freezing (HPF) to preserve the native structures. After undergoing the preparation steps of freeze substitution and EPON embedding, samples were finally sectioned and investigated in cLSM and SEM sequentially to get (see method and material). In total 15 EPON sections (100 nm each) were imaged in both microscopic modalities and the images from both microscopes were superimposed as previously described (Fig. 3a, Supplementary Fig. 3)^17^. CLEM images of each section were aligned in the correct order and eventually segmented for the reconstruction model with a volume of 1.5 µm (Fig. 3i). Each object was segmented in a different color for better distinction. Totally, 1446 BODIPY-labeled PS NPs were segmented in green (Fig. 3b, f) and they were found within 172 endocytotic vesicles (Fig. 3c, f). Furthermore, 20 endocytotic vesicles containing separated Cy5-labeled protein corona (Fig. 3e, f) were segmented within the PS-vesicles complex (Fig. 3c), and 271 objects of protein corona (Fig. 3d, f) were segmented according to CLEM images. In addition, 12 mitochondria (Fig. 3g, h) were found within the vesicle complex. Vesicles containing the separated protein corona and vesicles containing PS NPs were closely located together with mitochondria. Within the vesicles containing the separated protein corona, a few PS NPs were found.

### Separation of PS NPs and protein corona in the cell

**Fig. 4:**
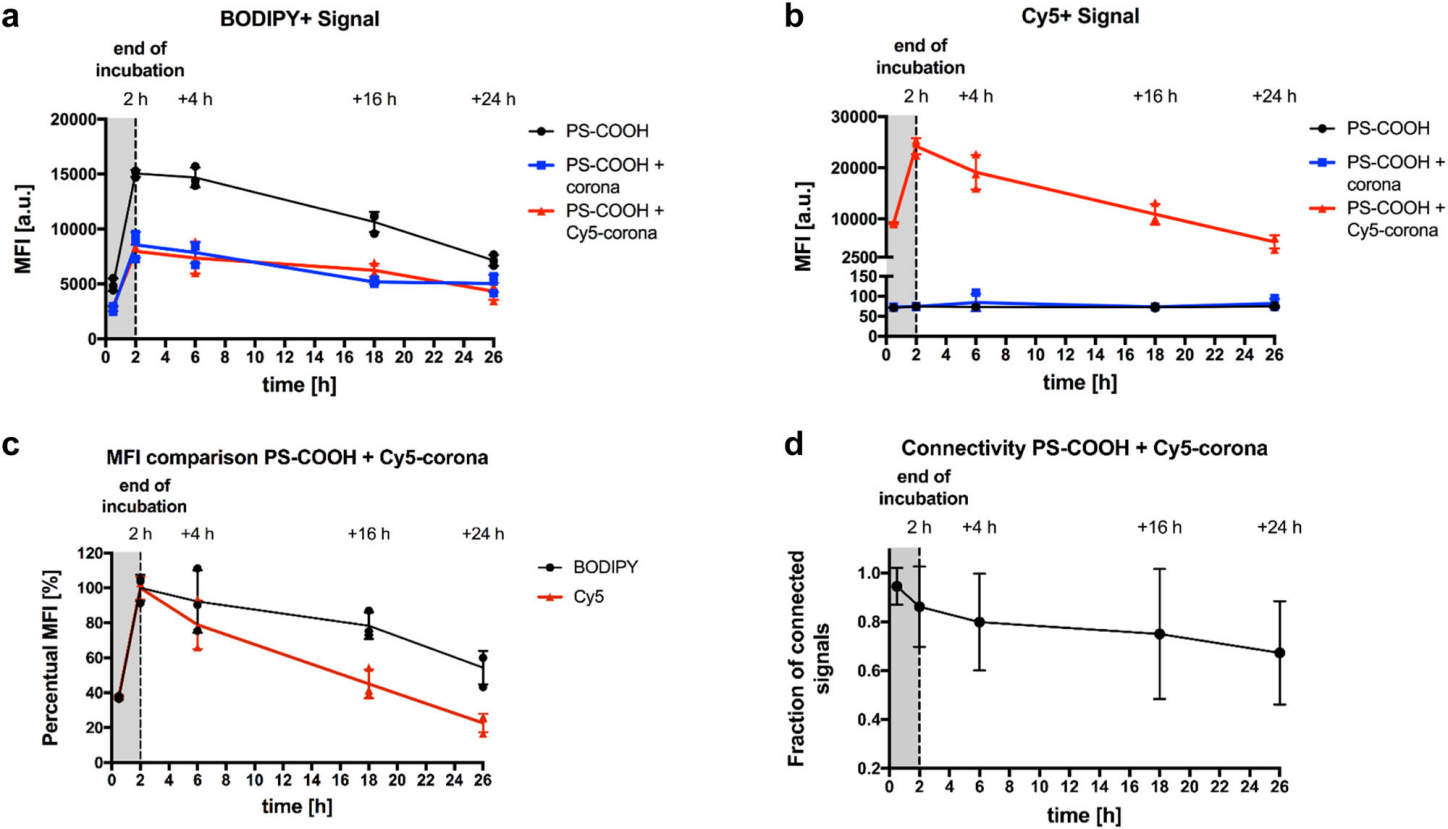
Flow cytometry and CLEM connectivity analysis reveal a slow separation of NP and protein corona after cell uptake. RAW264.7 cells were incubated with 150 µg mL^−1^ of carboxyl-functionalized PS NPs. Untreated NPs, NPs with an unlabeled protein corona, and NPs with a Cy5-labeled protein corona were used for the uptake experiment. The cells were incubated with the NPs for 2 h (dotted line). Subsequently, the NP-containing supernatant was removed and replaced with fresh culture medium without NPs. **a** Flow cytometry was performed to measure the median fluorescence intensity (MFI) of BODIPY (PS NPs). **b** The MFI of Cy5 (labeled corona proteins) was measured for the same events. The gating strategy for flow cytometry is provided in Supplementary Fig. 2. **c** The percentual MFI was plotted for the BODIPY and the Cy5 fluorescence in the case of the uptake of NPs with a Cy5-labeled corona. The percentual MFI value was calculated for each time point, comparing it to the highest MFI value measured (here: 2 h); data for **a**–**c** are shown as mean ± SD, *n* = 3. **d** The connectivity of BODIPY and Cy5 signals was analyzed for the uptake of NPs with a Cy5-labeled corona on CLEM images after different time points. The connectivity analysis was conducted by using an in-house ImageJ macro, calculating the fraction of connected signal intensities compared to the total intensities (data is shown as mean ± SD; *n*(0.5 h) = 28, *n*(2 h) = 67, *n*(2 h + 4 h) = 73, *n*(2 h + 16 h) = 14, *n*(2 h + 24 h) = 12).

We conducted additional flow cytometry analysis at different time points to investigate the fate of the internalized PS NPs and protein corona. For this, we retrieved the NPs-containing supernatant on RAW264.7 macrophages after an incubation time of 2 h and evaluated the BODIPY and Cy5 signal by flow cytometry after different additional incubation times. This procedure was performed for untreated PS NPs, PS NPs with an unlabeled protein corona, and PS NPs with a Cy5-labeled protein corona. Similar to the uptake analysis after 2 h, almost all measured cells were positive for BODIPY at every time point. If the cells were incubated with PS NPs with a Cy5-labeled protein corona we also detected that almost all measured cells were positive for Cy5 at every time point (Supplementary Fig. 4). Additionally, we evaluated the median fluorescence intensity (MFI) to understand the intensity of the signal after different incubation times and link this intensity to the fate of the NPs and the protein corona. Overall, the MFI for BODIPY decreased over time, indicating a lowering amount of PS NPs in the cells with time. This decrease can be partially explained by cell division, which was shown to reduce the amount of nanoparticles in a growing cell population over time^18^. However, the release of PS NPs was previously shown in different cell lines and contributes additionally to the signal decrease^19,20^. This decrease of PS NPs with time was observed for all three conditions of PS NPs (Fig. 4a). In general, the MFI of the samples with protein corona, unlabeled and Cy5-labeled showed a lower MFI than the PS NPs without protein corona. We observed a similarly reduced uptake for PS NPs with protein corona in past studies, which might be a result of adsorbed dysopsonin proteins^21^. The MFI for Cy5 showed a similar decrease over time for cells that were incubated with PS NPs with a Cy5-labeled protein corona compared to an unlabeled protein corona (Fig. 4b). To compare the decrease of the BODIPY and Cy5 signal in the case of the uptake of PS NPs with a Cy5-labeled protein corona, we calculated a percentual MFI. This percentual MFI is based on the normalization of the MFI value compared to the highest MFI value (the MFI value of 2 h). Interestingly, we observed that the MFI of Cy5 decreased faster than the MFI of BODIPY (Fig. 4c). Accordingly, we state that the macrophages metabolize or exocytose the fluorescent corona protein faster or process it by a different pathway than the PS NPs.

To support the flow cytometry findings, we analyzed the connectivity of the BODIPY and Cy5 signal on the embedded sections for CLEM (Fig. 4d). Here, we investigated multiple cells to generate reliable values. The connected signals were evaluated with an ImageJ macro (see method and material). In brief, the cell outlines were identified and defined on the section with cells with internalized PS NPs after different time points (Supplementary Fig. 5). Subsequently, thresholds were defined to identify the BODIPY signals (PS NPs) and Cy5 signals (protein corona; Supplementary Fig. 6) A protein corona object was counted as connected to a nanoparticle if its box overlapped or touched a box of a PS NP object. The intensities of the connected signals were then compared with the total signal intensities and a percentual fraction of connected signal intensity was calculated for each cell. We observed a decrease in the signal connectivity over the same time frame as in the flow cytometric experiment. Therefore, we conclude that the fluorescently labeled protein corona must be separated from the PS NP after the uptake. This separation is a process that takes several hours.

### The evolution of the protein corona after cellular uptake

**Fig. 5:**
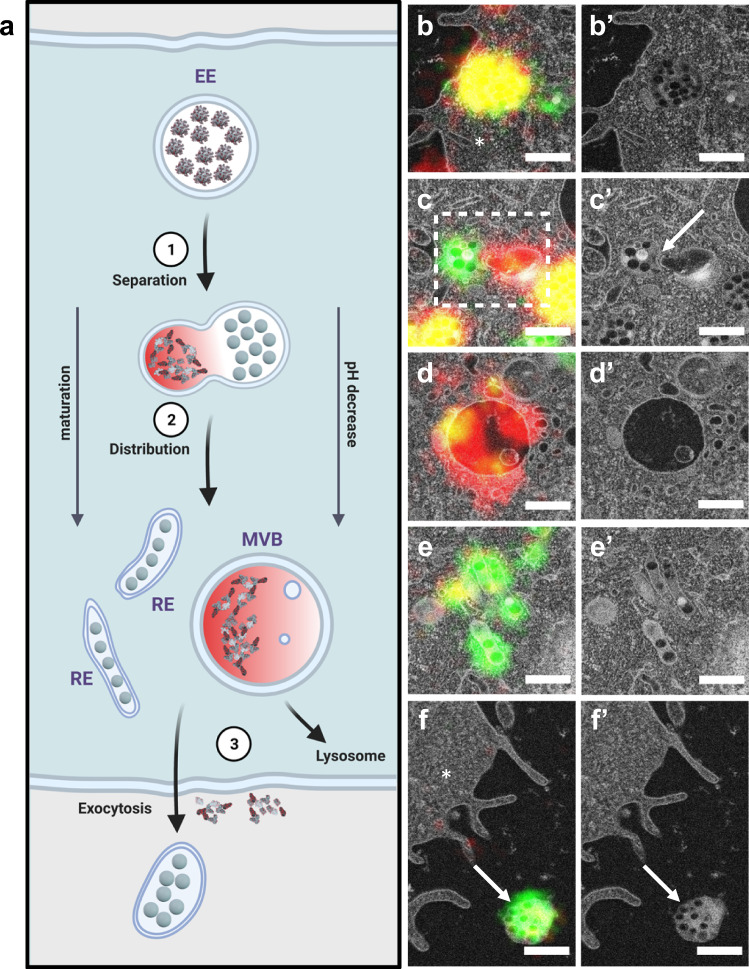
Intracellular fate of the protein corona and PS NPs illustrated by CLEM and EM micrographs. **a** Scheme of intracellular separation of the protein corona and PS NPs and exocytosis of NPs. Scheme was created with Biorender.com. **b**, **b**′ Protein corona-coated PS NPs in an endosome after internalization. Intracellular space is indicated with a star symbol. **c**, **c**′ Separation of the protein corona and PS NPs. Separation event is highlighted with a dotted square and an arrow. **d**, **d**′ Distribution of separated protein corona in an MVB. **e**, **e**′ Distribution of separated PS NPs in tubular recycling endosomes. **f**, **f**′ Exocytosis of separated PS NPs. Exocytosis of naked PS NPs is indicated by an arrow and intracellular space is indicated with a star symbol. Red represents the Cy5-labeled protein corona, green represents BODIPY-labeled PS NPs, yellow represents the overlay of the protein corona and PS NPs. Scale bars: 500 nm.

**Fig. 6:**
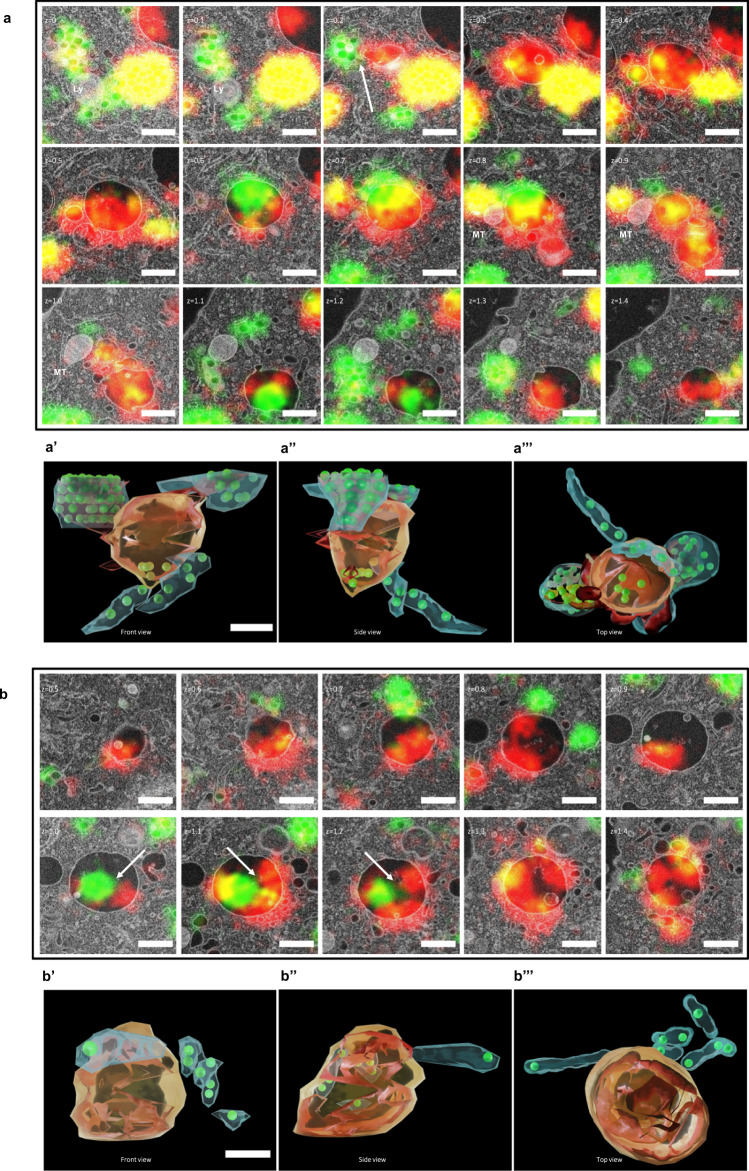
Volume CLEM and segmented models of the separation of the protein corona and PS NPs. PS NPs with Cy5-labeled protein corona were incubated with RAW264.7 cells and imaged in CLEM after 2 h + 24 h. **a** Volume CLEM of the separation of protein corona and PS NPs in endosomes. **a**′ Front view of the segmented model in **a**. **a″**. Side view of the segmented model in **a**. **a**‴. Top view of the segmented model in **a**. **b**. Volume CLEM of an MVB with separated protein corona and PS NPs. **b**′. Front view of segmented model in **b**. **b**″. Side view of segmented model in **b**. **b**‴. Top view of segmented model in **b**. Red represents the Cy5-labeled protein corona, green represents BODIPY-labeled PS NPs, yellow represents the overlay of the protein corona and PS NPs. Scale bars: 500 nm.

The intracellular fate of the protein corona and PS NPs was further revealed by the serial CLEM images. The protein corona (Cy5, red) and PS NPs (BOPDIPY, green) were mostly localized in round endosomes (Fig. 5b, b′, yellow), whereas the separated protein corona signal was exclusively found in multivesicular bodies (MVBs) (Fig. 5d, d′, MVBs in Supplementary Fig. 7, red). These events were repeatedly observed in various cells (Supplementary Fig. 8). In fact, the protein corona was exclusively observed within vesicular boundaries at 2 h and 2 h + 24 h (Supplementary Fig. 9), which excludes the Cy5-labeled proteins translocating to the cytosol or to other compartments outside the endo-lysosomal system during the trafficking. We also localized elongated tubular vesicles around MVBs containing only PS NPs. These tubular vesicles can be morphologically identified as recycling endosomes (REs) (Fig. 5e, e′)^22^. We confirmed that PS NPs without protein corona were also transported to REs (Supplementary Fig. 10). Upon the observation that the protein corona and PS NPs were distributed separately in morphologically different endosomes, we further identified the sorting and separation event (Fig. 5c, c′) by investigating the CLEM images where two signals were closely connected but not overlapped. Depending on the EM micrograph (Fig. 5c′), the endosome containing the protein corona exhibited a ruffling towards the endosome containing PS NPs (Fig. 5c′, white arrow), indicating a possible division and thus formation of two distinctly loaded endosomal vesicles. Meanwhile, the endosome that contained only the protein corona (Fig. 5c, red) was found to be developed into an MVB in later sections (Fig. 6a). This finding confirms the earlier observation of the separated protein corona. Additionally, we observed exocytosis of separated PS NPs (Fig. 5f, f′) in another cell (Supplementary Figs. 12 and 13), implying that PS NPs could be exocytosed by REs after being separated from the protein corona. To further demonstrate the separation event and the distribution of separated protein corona in MVBs, serial CLEM images of areas in Fig. 5c, d and were shown in detail accordingly (Fig. 6a, b). The segmented models (Fig. 6a′, a″, a‴) of Fig. 6a showed the close spatial relationship between an endosome containing separated PS NPs (green) and an MVB containing the separated protein corona (red). An endosome with protein corona-coated PS NPs and tubular REs with separated PS NPs were located next to the MVB indicating the separation might be an ongoing process. The segmented model (Fig. 6b′, b″, b‴) of Fig. 6b revealed dimensionally that several REs containing separated were located close to the MVB containing separated protein corona. The spatial information from both models further hinted that the separation and distribution of protein corona and PS NPs were closely related to the functions of different endosomes.

According to our CLEM results, the protein corona-coated PS NPs were mainly located in crowded round-shaped endosomes, whereas the separated protein corona was exclusively located in MVBs, and the separated PS NPs were found in tubular REs (Supplementary Figs. 7 and 8). Because our flow cytometry results showed the successful co-internalization of the protein corona and NPs, we assume that the endosomes with the protein corona-coated NPs were the primary or early endosomes (EEs). At the same time, it is known that EEs are crucial for sorting functions^22–24^ and are able to generate tubular REs for further transportation or recycling^22,25^. Therefore, we consider that the separation or sorting of the protein corona and PS NPs starts at the site of early endosomes when REs containing PS NPs begin to form while the protein corona begins to separate from NPs. Furthermore, we observed the separated protein corona only in large MVBs containing intraluminal vesicles^26,27^. These MVBs mature from EEs^26^ and more specifically from the main bodies of the EEs after the formation of REs^23,24^. We hypothesize that during the maturation of the EEs after internalization, NPs start to separate from the protein corona and are transported into REs. As the maturation of the EEs into MVBs completes, most NPs are then transported into REs leaving behind the protein corona within the matured MVBs (Fig. 5a). This hypothesis correlates with the endosome maturation during the endocytic pathway and is supported by our CLEM results. Exocytosed NPs within a vesicle were also observed at the same condition (Supplementary Fig. 12), and this phenomenon has been reported in different cell lines^19,20^. We speculate that the exocytosis might happen via the recycling of REs to the plasma membrane after protein corona separation. On the other hand, depending on the MVB maturation, the separated protein corona in the MVBs might eventually accumulate in lysosomes^27^ or exocytose via exosomes^28^. Degradation of the protein corona in lysosomes is predictable and was previously observed^16^. Nevertheless, we have captured the Cy5 signals around the plasma membrane, either co-localizing with some membranous vesicles or on the membrane ruffling (Supplementary Fig. 11). These Cy5 signals outside of the cell might be the evidence of the exocytosed protein corona from MVBs and suggest an alternative destiny of the separated protein corona.

We can point out various potential reasons for the separation of the protein corona from the NPs. One reason behind the separation of the protein corona and NPs might be related to the changing acidification within different endosomes. To investigate the role of the acidification, we conducted a fluorescence cross-correlation spectroscopy (FCCS) evaluation of the stability of the Cy5-labeled protein corona on the PS NPs in cell culture medium at different pH values. The FCCS results suggested that the protein corona on NPs remained stable in the range of pH 7.6-pH 5.5. At pH 4.5 the Cy5 and BODIPY signals separated over a time span of 24 h indicating disintegration of the protein corona (Supplementary Fig. 14). The pH value in different endosomes decreases, thus the endosomes acidifies, along the endocytic pathway during the endosomal maturation^23,29^. It is well understood for in vitro situations that the composition and stability of the protein corona are strongly affected by the environmental pH^30,31^. We suggest, that due to the decreased pH from EEs to MVBs, the proteins on the protein corona might lose their affinity to the surface of the NPs because the evolution of protein corona is dynamic under a fickle environment^32,33^. Additionally, the acidification might cause conformation changes of the adsorbed proteins^30,34^ which can consequently influence and alter protein-protein and most protein-NP interactions of the protein corona. However, our in vitro experiment showed a disintegration at pH 4.5 and not at higher pH values (Supplementary Fig. 14). Particularly, endosomes before the late-endosomal stage show pH values between 6.5 and 5.5^35^. Therefore, acidification can only be seen as one possible reason for the separation of the fluorescent-labeled protein corona from the PS NPs.

Furthermore, the protein corona-coated NPs transit through dynamic biological environments, being subjected to changes of the ionic environment and being confronted with new, intracellular proteins which were not originally present in the plasma. Endosomal maturation involves a constant efflux of cations, such as Ca^2+^, Na^+^, and K^+^, and an influx countering Cl^-^ anions^36^. The importance of the ionic environment for the formation of the protein corona was previously explored^37^, making it possible that continuous changes in the ionic environment could contribute to the displacement or rearrangement of the protein corona. Other studies highlighted the dynamic behavior of the protein corona after transitioning through different biological fluids^14^ and within cells^38,39^. It is possible that intracellular proteins exhibit a higher affinity towards the NPs’ surface and, therefore, adsorb on the NPs’ surface intracellularly and replace previous proteins, leading to a newly formed protein corona. Additionally, protein degradation via late-endosomal proteases is likely to contribute to the identity change of the protein corona on NPs if they surpass the early endosomal stage^16^. Thus, this highly dynamic molecular environment might further contribute to the separation of the fluorescently labeled plasma proteins from the corona.

Ultimately, with our work we identified the separation process in a time-dependent manner with the strong support of high-resolution images. Nevertheless, future investigations must uncover the exact molecular aspects behind the separation. Here, we see potential in stable isotope-labeling of amino acids to precisely track the protein corona and related peptides by degradation in the intracellular environment. In addition, these studies will profit from staining intracellular compartments, e.g. by transfection or antibodies, and including state-of-the-art microscopic techniques, such as cryogenic electron microscopy (cryo-EM) for structural studies or super-resolution live imaging.

Our results demonstrate the progressive separation of PS NPs from an associated protein corona that occurs over a timeframe of several hours. We visualized these events within the cell by volume CLEM and propose an intracellular pathway for the hereby used nanoparticular system that includes (1) the mutual internalization into the endosomal system, (2) the separation of NPs and the protein corona by endosomal sorting mechanisms, (3) the presence of NPs in elongated REs and the protein corona in MVBs, and (4) the exocytosis of PS NPs and while the protein corona remains in the endo-lysosome. However, all these findings are based on the examination of one cell line. But in order to be able to make a general statement on the intracellular processing of the protein corona, it is of course necessary to examine a wide variety of different cell lines.

With these findings, we should ultimately be able to understand engineered artificial protein coronas on NPs and optimize the release of the API towards a more sophisticated design of nanotherapeutics.

## Methods

### Synthesis of carboxy-functionalized polystyrene nanoparticles

A macroemulsion was prepared with a continuous phase containing 600 mg Lutensol AT50 (BASF, Germany) solution in 24 mL Milli-Pore water as surfactant and a dispersed phase containing 5.88 g distilled styrene, 251 mg hexadecane (Acros Thermo Fisher, Germany) as hydrophobe, 153 mg distilled acrylic acid for the introduction of carboxy-functionalities, 6.1 mg BODIPY methacrylate as fluorescent dye and 100 mg 2,2′azobis(2methylbutyronitrile) V59 (Wako, Germany) as oilsoluble azo initiator.

The dispersed phase was mechanically stirred. The continuous phase was slowly added to the dispersed phase to achieve homogenization. The macroemulsion was stirred for 1 h at the highest speed. The macroemulsion was then ultrasonicated with a Branson Sonifier (1/2″ tip, 6.5 nm diameter) for 2 min at 450 W 90% amplitude under ice cooling to obtain a miniemulsion. The miniemulsion was transferred into a 50 mL flask and heated to 72 °C in an oil bath under stirring. The polymerization was performed for 11 h. Subsequently, the dispersion was centrifuged for 1.5 h at 15,500 × *g*, 5 times for purification. The supernatant was removed after each centrifugation and the pellet redispersed in Milli-pore water. Nanoparticles were characterized by dynamic light scattering for the average diameter. The zeta potential was measured by diluting particles in a 1 mM potassium chloride solution. Dynamic light scattering and zeta potential were measured by a Malvern Zetasizer nano-s90 (Malvern Instruments, Germany).

### Fluorescent labeling of murine plasma proteins with Cy5

Cyanin5(Cy5)-NHS ester (Lumiprobe, Germany) was added to murine plasma (GeneTex, USA) with a molar excess of 1.6 to one amino group, assuming all proteins in the plasma to be serum albumin. The reaction was carried out for 1 h at room temperature, shaking. Purification of labeled proteins and removal of unreacted free dye was performed with Zeba^TM^ Spin 7 kDa MWCO columns (Thermo Fisher, Germany), following the manufacturer’s instructions.

### Protein corona preparation

Protein corona preparation was performed according to a previously published protocol^40^. Briefly, to form the protein corona, 1 mg of carboxy-functionalized polystyrene nanoparticles, which corresponds roughly to a surface of 0.01 m^2^, was added to 1 mL of unlabeled or Cy5-labeled murine plasma and incubated at 37 °C for 1 h, shaking. After the incubation, samples were centrifuged at 20,000 × *g* for 30 min at 4 °C (5804R, Eppendorf, Germany), the supernatant removed and the pellet resuspended in 1 mL PBS (Sigma-Aldrich, Germany). To remove non-adsorbed and low-affinity proteins, one wash step was performed for cellular uptake experiments and three wash steps for protein analysis. For protein analysis, proteins were desorbed with 100 μL of desorption buffer (2% (w/v) SDS, 62.5 mM Tris-HCl) after incubation at 95 °C for 5 min, shaking and one centrifugation step as described above.

### Protein quantification

The Protein concentration was quantified by Pierce^TM^ 660 nm Protein Assay Reagent (Thermo Scientific, Germany) following the manufacturer’s instructions. Ionic Detergent Compatibility Reagent (Thermo Scientific, Germany) was added to the assay reagent to measure samples containing SDS. A standard calibration curve was prepared with bovine serum albumin (Sigma-Aldrich, Germany). Absorption was measured with an Infinite M1000 plate reader (Tecan, Switzerland) at 660 nm.

### SDS-PAGE, silver staining, and in-gel fluorescence detection

To perform SDS-PAGE, 2 µg of protein was diluted with deionized water to a volume of 26 μL. To this sample dilution, 4 μL of NuPAGE^TM^ Sample Reducing Agent and 10 μL of NuPAGE^TM^ LDS Sample Buffer (both Invitrogen, Germany) were added and incubated at 70 °C for 10 min for protein denaturation. The samples were loaded on a Bolt^TM^ 10% Bis-Tris Plus gel using NuPAGE^TM^ MES SDS Running Buffer (both Invitrogen, Germany). Protein electrophoresis was run for 1 h at 200 V. SeeBlue^TM^ Plus2 Pre-Stained Standard (Invitrogen, Germany) was used as a molecular weight marker. SilverQuest^TM^ Silver Staining Kit (Invitrogen, Germany) was used to stain the gels according to the manufacturer’s instructions. Pictures of the developed gels were taken with a View Pix 1100 scanning system (Biostep, Germany). The in-gel fluorescence was detected by an IVIS Spectrum CT (PerkinElmer, USA) with an excitation wavelength of 650 nm, an emission wavelength of 680 nm, and an exposure time of 3 s. The measurements were conducted in duplicates with similar results.

### In-solution tryptic digestion

Proteins were forwarded to an in-solution tryptic digest for LC–MS measurements, which was performed in previous studies^15,41^. Before starting the digestion protocol, SDS was removed from samples with Pierce^TM^ Detergent Removal Spin Columns (Thermo Scientific, Germany) according to the manufacturer’s instructions. After SDS removal, 25 µg of protein per sample were precipitated with ProteoExtract Protein Precipitation Kit (CalBioChem, Germany), following the manufacturer’s instructions. The precipitated proteins were isolated by centrifugation at 10,000 × *g* for 10 min at RT and two wash steps. The supernatant was finally removed and the pellet dried for 5–10 min. To solubilize the dried protein pellet, 0,1% RapiGest SF surfactant (Waters Corporation, Germany), dissolved in 50 mM ammonium carbonate buffer was added and incubated at 80 °C for 15 min. A dithiothreitol (Sigma, Germany) solution was added in a final concentration of 5 mM to reduce protein disulfide bonds. The reaction was run at 56 °C for 45 min. To alkalize the proteins, 500 mM iodoacetamide (Sigma, Germany) solution was added for a final concentration of 15 mM. The reaction was run at RT for 1 h in the dark. To initiate digestion, a trypsin (Promega, Germany) solution was added in the mass ratio of 50: 1 (protein: trypsin). After digestion for 16 h at 37 °C, 2 µL of hydrochloric acid (Sigma, Germany) was added to stop the digest and incubated further for 45 min at 37 °C. Finally, the aggregated degradation products were removed by centrifugation at 13,000 × *g* for 15 min at 4 °C. The supernatant containing peptides was transferred into new tubes.

### Liquid chromatography coupled to mass spectrometry (LC–MS)

Digested samples were diluted with LC–MS grade water (Merck, Germany) containing 0.1% formic acid (Sigma, Germany). To perform an absolute quantification according to a previously established protocol^42^, samples were additionally spiked with 50 fmol μL^−1^ HI3 Ecoli Standard (Waters Corporation, Germany). The samples were measured by a nanoACQUITY UPLC system, installed with a C18 nanoACQUITY trap column (5 μm, 180 μm × 20 mm) and a C18 analytical reversed-phase column (1.7 μm, 75 μm × 150 mm; all Waters Corporation, Germany). The mobile phases used for separation consisted of (A) 0.1% (v/v) formic acid in water and (B) 0.1% (v/v) formic acid in acetonitrile (Biosolve, Germany). A gradient of 2% to 37% of mobile phase B over 70 min was performed. The samples flow rate was set to 0.3 μL min^−1^ and the flow rate of the referent components Glu-Fibrinopeptide and LeuEnkephalin (both Sigma, Germany) were set to a flow rate of 0.5 μL min^−1^. The nanoACQUITY UPLC system was connected to a Synapt G2Si mass spectrometer (Waters Corporation, Germany) with electrospray ionization (ESI). The NanoLockSpray source was set to positive mode. The measurements were performed in resolution mode and data-independent acquisition (MS^E^). Following settings were used: mass to charge range of 50–2000 Da, scan time of 1 s, ramped trap collision energy from 20 to 40 V, and data acquisition of 90 min. Technical replicates were measured for each sample. The system was operated by the software MassLynx 4.1 (Waters Corporation).

### Protein identification

Measured peptides and assigned proteins were processed with the software Progenesis QI 2.0 (Nonlinear Dynamics). The settings and procedure were described in previous studies^6^. Briefly, we defined the noise reduction threshold for low energy, elevated energy, and peptide intensity as 120, 25, and 750 counts, respectively. The murine proteome database with reviewed proteins was retrieved from uniport (swiss prot). The protein sequence information for the standard protein, Hi3 E. coli standard, was added to perform absolute quantification. The identification runs were performed with the following settings: one missed cleavage, maximum protein mass of 600 kDa, fixed carbamidomethyl modification for cysteine, variable oxidation for methionine, a minimum of three assigned fragments per peptide, a minimum of two assigned peptides per protein, a minimum of five assigned fragments per protein, and a score parameter below 4. A table with all identified proteins is provided separately in the supplementary information.

### RAW264.7 cell culture

The murine macrophage cell line RAW264.7 was cultivated with Dulbecco’s Modified Eagle Medium, supplemented with 10% FBS, 100 U mL^−1^ penicillin, and 100 mg mL^−1^ streptomycin (all Gibco/Thermo Fisher, Germany). The cells were cultured in an incubator (CO_2_ Incubator C200, Labotect, Germany) at 37 °C, 5% CO_2_, and 95% relative humidity. For subculturing and harvesting, RAW264.7 cells were briefly washed with PBS prior to adding 0.25% Trypsin-EDTA (Gibco/Thermo Fisher, Germany) for detachment. Cells were collected after incubation for 5 min at 37 °C, 5% CO_2_, and 95% relative humidity. The same volume of cell culture medium was added and the cells were sedimented by a centrifugation step of 300 × *g* for 5 min. The supernatant was removed. The cell viability and count were measured by an automated cell counter (TC10, Bio-Rad, Germany). The cells were diluted in cell culture medium for the next passage or experiment.

### Cell uptake analysis with flow cytometry

After harvesting the cells, 150,000 RAW264.7 cells were seeded per well in a 24 well plate. To induce cell attachment, cells were incubated at 37 °C and 5% CO_2_ overnight. On the next day, the medium was removed and the cells were washed once with 1 mL of PBS. Carboxy-functionalized PS NPs were diluted to a concentration of 150 µg mL^−1^ in DMEM without FBS and added to the cells in a volume of 200 µL. For a control experiment, Cy5-labeled murine plasma proteins were added in a comparable amount to the protein corona to the cells, which corresponded to 9 µg mL^−1^. Uptake after different incubation times was measured by flow cytometry. To harvest the cells, the culture medium was removed and the cells washed once with 1 mL of PBS. Subsequently, 250 µL 0.25% Trypsin-EDTA was added and the cells incubated for 5 min at 37 °C and 5% CO_2_ to induce detachment. After detachment, 250 µL DMEM without FBS was added and the cells were transferred to 1.5 mL tubes. The cells were centrifuged at 300 × *g* for 5 min. The supernatant removed and the cells resuspended in 1 mL of PBS.

The flow cytometry measurements were performed with an Attune NxT (Thermo Fisher, Germany). To detect BODIPY (Carboxy-functionalized PS NPs) a 488 nm excitation laser was employed with a 530/30 nm band-pass filter. To detect Cy5 (labeled murine plasma proteins) a 638 nm excitation laser was used with a 670/14 nm band-pass filter. Cells were analyzed by forward scatter and sideward scatter to discriminate cellular debris and identify the cell population. Next, fluorescence properties of the identified cell population were analyzed as the percentage of gated fluorescent events or as the median fluorescent intensity (MFI). Processing of flow cytometry data was performed with Attune NxT Software (Thermo Fisher, USA). Data for flow cytometry is presented as mean ± SD, *n* = 3.

### Fluorescence cross-correlation spectroscopy (FCCS)

Protein corona preparation was performed as described above with one wash step to be comparable with the cell experiments. After the wash step, the PS NPs with the Cy5-labeled protein corona were resuspended in DMEM with different pH values (all without FBS). Hydrochloric acid (1 N, Honeywell^TM^ Fluka^TM^, Germany) was used to adjust the pH value of DMEM. The pH values were measured with a pH electrode (Lab 855, SI Analytics, Germany). The stability of the Cy5-labeled protein corona on the BODIPY-labeled PS NPs was studied by performing FCCS experiments^43,44^ at different time intervals at the respective pH. An eight-well polystyrene, chambered cover glass (Laboratory-Tek, Nalge Nunc International) was used as a sample cell. The FCCS experiments were performed on a commercial setup (LSM 880, Carl Zeiss, Jena, Germany). For excitation of the BODIPY and Cy5-labeled species an argon ion laser (488 nm) and a He/Ne-laser (633 nm) were used respectively. The excitation light was focused into the studied solution by a high numerical aperture water immersion objective (C-Apochromat 40x/1.2 W, Carl Zeiss, Jena, Germany). The fluorescence was collected with the same objective and after passing through a confocal pinhole, directed to a spectral detection unit (Quasar, Carl Zeiss). In this unit, emission was spectrally separated by a grating element on a 32 channel array of GaAsP detectors operating in a single photon counting mode. The emission of BODIPY was detected in the spectral range 500–550 nm and that of Cy5 in the range 640–700 nm. The recorded experimental auto- and cross-correlation curves were fitted with the theoretical model function for freely diffusing fluorescence species^43,44^. The fits yielded the diffusion coefficients and the concentrations of the BODIPY labeled, Cy5 labeled and double labeled species. To evaluated the stability of the protein corona on the PS NPs, we calculated the ratio of the concentration of the Cy5 labeled species (all plasma proteins) to the concentration of the double labeled species (plasma proteins on the PS NPs) and monitored the time dependence of this ratio (Fig. 4).

### Data representation

GraphPad Prism 8 (GraphPad Software, USA) was utilized for data visualization. The data are shown as means ± standard deviation (SD) of the values.

### Correlative light and electron microscopy (CLEM) sample preparation

RAW264.7 macrophages were seeded onto 3 mm sapphire disks (M. Wohlwend GmbH, Switzerland). Sapphire disks were pre-coated with a 10-nm-thick carbon layer using an EM MED020 instrument (Leica, Germany). The coated sapphire disks were dried and sterilized in an oven at 120 °C overnight before use. Macrophages were seeded onto sapphire disks in 12-well plates overnight for cell attachment. Nanoparticles were added in a concentration of 150 µg mL^−1^ to the macrophages and incubated in a humidified incubator at 37 °C and 5% CO_2_. The incubation time points are indicated for each figure showing CLEM images. After the incubation, each sapphire disk was collected from the 12-well plates and slightly immersed into 1-hexadecene before placing them between two aluminum plates (3 mm, Plano). The aluminum ‘sandwich’ structure was placed into a specimen holder for high pressure freezing in a Wohlwend HPF Compact 01 high pressure freezer with a pressure of 2100 bar for 2–3 s. The specimen holder was withdrawn from the freezer and immersed into liquid nitrogen to release the sample. The frozen sample was then labeled and stored in a container filled with liquid nitrogen.

Frozen sapphire discs were carefully removed from the aluminum ‘sandwich’ and transferred into 1 mL pre-cooled freeze substitution medium (0.2% (w/v) osmium tetroxide, 0.1% (w/v) uranyl acetate, 5% (v/v) distilled water in acetone) and kept in a freeze substitution unit (AFS2, Leica, Germany). Samples were then slowly warmed up to 0 °C over a period of 20 h in the unit. After being warmed up, the freeze-substituted samples were brought to room temperature, then the substitution medium was removed and the discs were washed 3 times with acetone at half an hour intervals. Then the discs were infiltrated sequentially in gradient epoxy resin-acetone mixture (1;1, 1:2, and 2:1) for 1 h. Samples were then infiltrated in 100% epoxy resin overnight. Finally, each sample was transferred into a new Eppendorf tube containing fresh epoxy resin for polymerization at 60 °C for 24 h.

### Serial sectioning and CLEM imaging

After polymerization, sapphire discs were detached using liquid nitrogen. Afterward, these resin blocks were trimmed for later serial sectioning. Trimmed resin blocks were sectioned using an ultra 45° Jumbo diamond knife (Diatome, Switzerland) with a home-made water draining setup. An ITO coated coverslip (SPI) was placed and fixed at the other end of the diamond knife. Diluted glue was placed on either top or bottom side of the trimmed resin block to make sure the sections stay attached in the correct order while sectioning. The section band was carefully moved to the coverslip with an eyelash until one end of the band touched the coverslip. Afterwards, the water level was carefully lowered with the draining setup until the whole section band was slowly attached to the coverslip. The coverslip was then placed on a preheated hotplate to dry completely. The dried coverslip was then imaged in cLSM (SP5, Leica, Germany) with a 20 × 0.75 NA dry objective for overviews or 63 × 1.4 NA oil immersion objective to capture images of selected areas. At least 3 different areas have been imaged yielding similar results. The same coverslip was later mounted onto a clamp holder to be imaged in a HITACHI SU8000 with 2.5 kV landing voltage, 15 mA probe current and using the HA-BSE detector. Image registration and alignment of light and electron microscopy images were accomplished in Fiji and icy with the eC-CLEM plugin.

### Fluorescence connectivity measurement

To identify the separation of the Cy5-labeled protein corona from the PS NPs, we developed a macro that measures the connectivity of red and green signals using ImageJ. The ijm file of the used ImageJ macro is included in the supplementary information. We evaluated 12–73 cells per time point. The outlines of the cells were defined manually in the reflected light channel or EM images. In some cases, several adjacent images were used to include enough cells for the evaluation. Thresholds were defined to identify NP and protein corona objects and their signal intensities were evaluated using the ParticleAnalyzer plugin. A protein corona object was counted as connected to a PS NP object if its box overlapped or touched a box of PS NP object. The connected signal intensity of the connected protein corona were was compared with the total signal intensity of protein corona for each cell and a percentual fraction of connected signal intensity was calculated. The connectivity was determined as the mean for all cells in a region of adjacent cells having at least five green and three red objects. The cell-based standard deviation was plotted with the mean of the connectivity.

### Reporting summary

Further information on research design is available in the Nature Portfolio Reporting Summary linked to this article.

## Supplementary information

Supplementary Information

Reporting Summary

## Data Availability

The data generated in this study are provided in the Supplementary Information and the Source Data file provided with this paper. Data is also available from the corresponding author upon request. Source data are provided with this paper.

## Source data

Source Data

## Code availability

The ImageJ code for the evaluation of the connectivity is provided in the Supplementary Information.
