# Supplementary material for: Endosomal sorting results in a selective separation of the protein corona from nanoparticles
Source: Nat Commun. 2023 Jan 18;14:295. doi: 10.1038/s41467-023-35902-9 (PMC9847456; doi:10.1038/s41467-023-35902-9)
Supplement: Supplementary file 1 — Supplementary Information [file 41467_2023_35902_MOESM1_ESM.pdf]

Supplementary information

Endosomal sorting results in a selective separation of the protein corona from nanoparticles

Shen Han [1,‡], Richard da Costa Marques [1,2,‡], Johanna Simon [1,2,‡], Anke Kaltbeitzel [1], Kaloian Koynov [1], Katharina Landfester [1], Volker Mailänder [1,2,§], Ingo Lieberwirth [1,§*]

[1] Max Planck Institute for Polymer Research, Ackermannweg 10, 55128 Mainz, Germany.

[2] Dermatology Clinic, University Medical Center of the Johannes Gutenberg-University Mainz, Langenbeckstr. 1, 55131 Mainz, Germany.

[‡] These authors conributed equally

[§] These authors jointly supervised this work

*Corresponding author

Supplementary Figures

Supplementary Table 1: Characterization of carboxy-functionalized polystyrene nanoparticles. The used surfactant, fluorescent dye with excitation and emission wavelength, average nanoparticle diameter D_Z, and zeta potential ζ are listed. The average diameter was measured by dynamic light scattering. The average diameter and zeta potential were measured by a Malvern Zetasizer nano-s90 .

Name	Surfactant	Fluorescent Dye λ (Excitation/Emission) [nm]	D_Z [nm]	ζ [mV]
PS-COOH	Lutensol	BODIPY (523/536)	116	-7.21

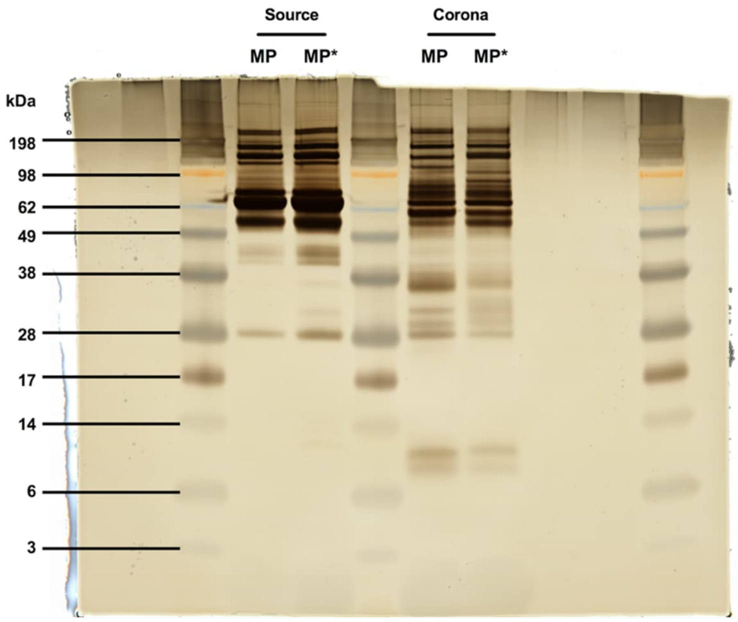

Supplementary Figure 1: SDS-PAGE of murine plasma and protein corona samples. Unlabeled murine plasma (MP), Cy5-labeled murine plasma (MP*), and associated protein corona samples were analyzed by SDS-PAGE and silver staining. Corona proteins were obtained after incubation of carboxyl-functionalized PS NPs in plasma, washing, and desorption with 2% of SDS.

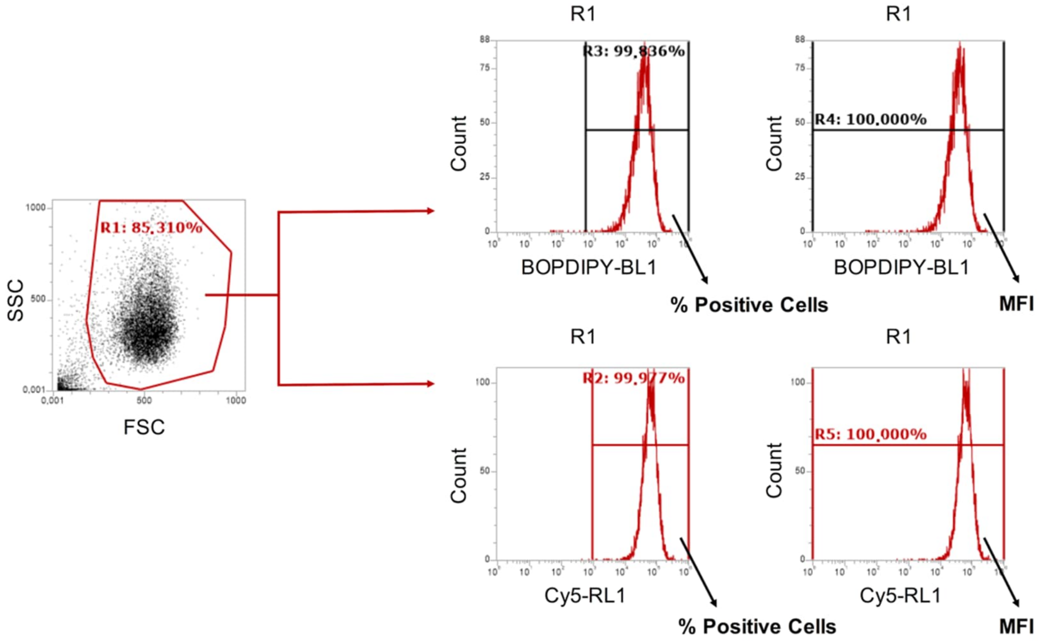

Supplementary Figure 2: Gating strategy of the flow cytometry experiments. RAW264.7 cells were analyzed by forward scatter and sideward scatter to identify the cell population of interest and exclude cell debris. The cell population of interest was gated (R1). The events of the gate R1 were analyzed for their fluorescence of BODIPY of the PS NPs with the channel BL-1 (excitation: 488 nm, band-pass filter: 530/30 nm) and of Cy5 of the labeled protein corona with the channel RL-1 (excitation: 638 nm, band-pass filter: 670/14 nm). The percentage of BODPIY- and Cy5-positive cell populations were identified with a histogram by comparison with a cell-only control. The MFI was measured for all events of R1.

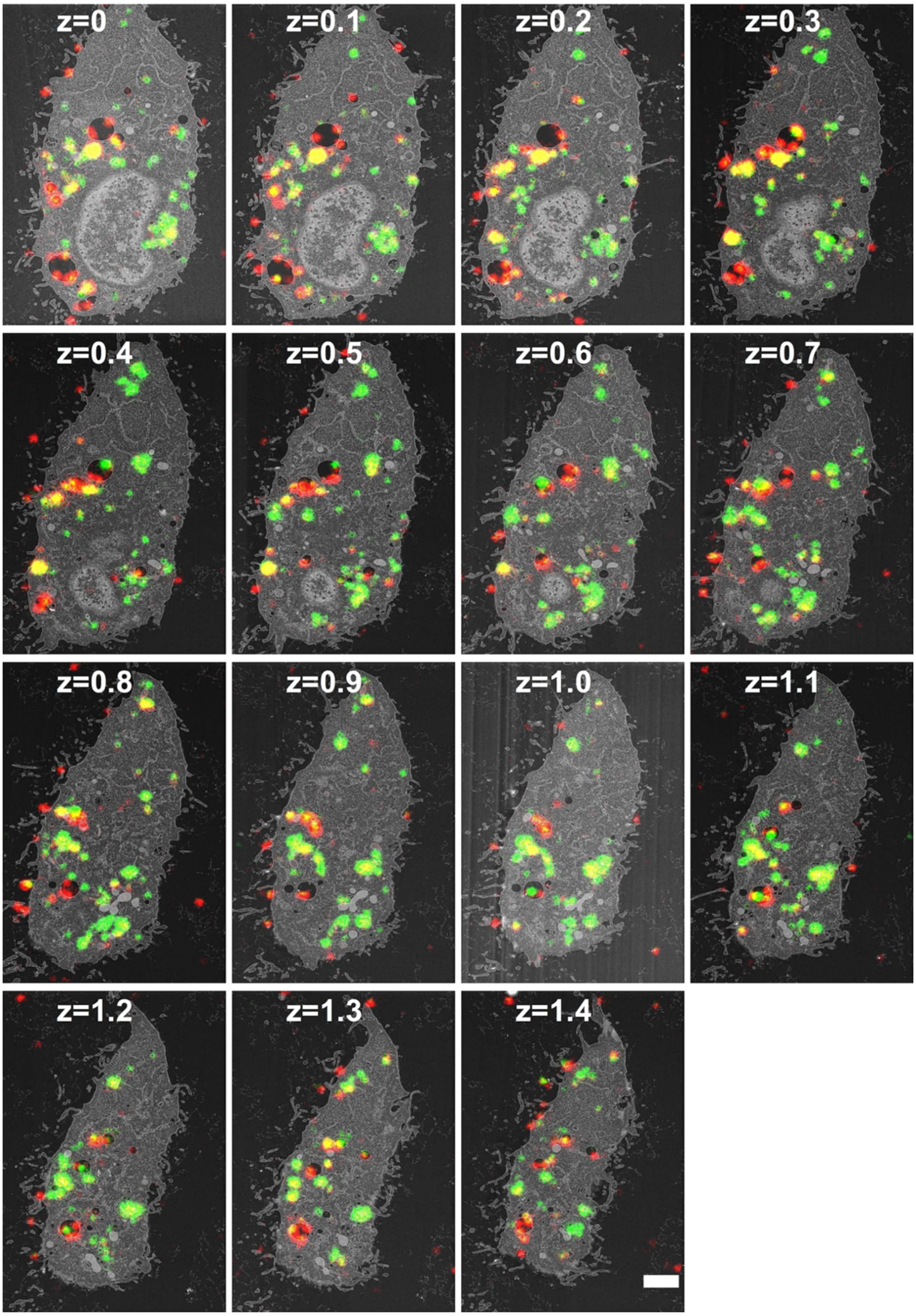

Supplementary Figure 3: Volume CLEM of a RAW macrophage with internalized PS NPs with Cy5-labeled protein corona. PS NPs with Cy5-labeled protein corona were incubated with RAW264.7 cells and imaged in CLEM after 2 h + 24 h. z represents the relative depth of each section in µm. Red represents the Cy5-labeled protein corona, green represents BODIPY-labeled PS NPs, yellow represents the overlay of the protein corona and PS NPs. Scale bars: 2 µm.

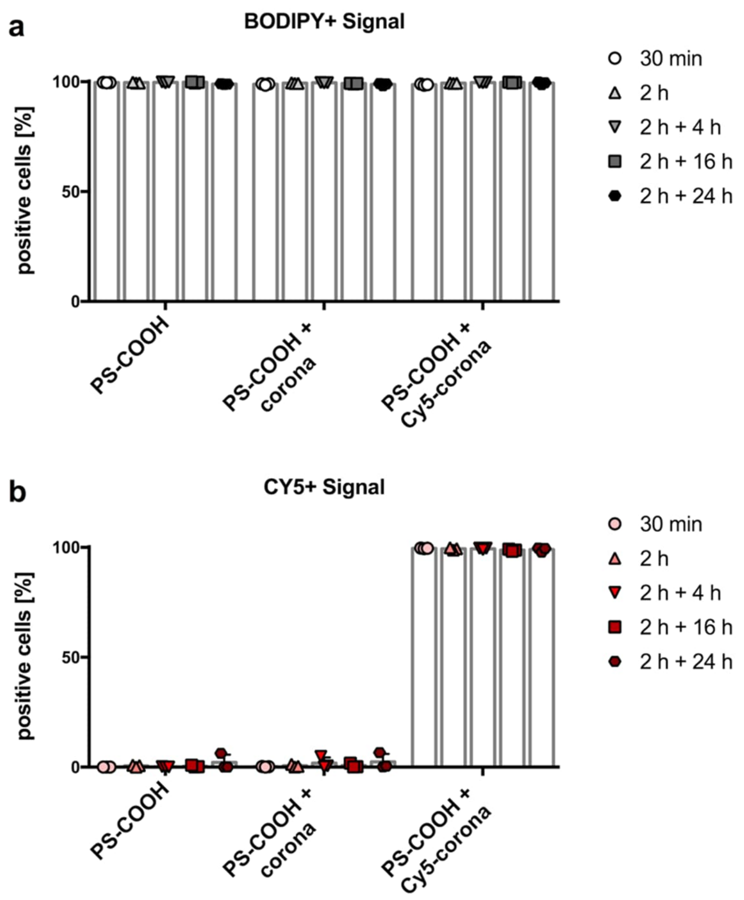

Supplementary Figure 4: Murine macrophages show a very high uptake of differently treated NPs after various time points. RAW264.7 cells were incubated with 150 µg mL^{-1} of carboxyl-functionalized PS NPs. Untreated NPs, NPs with an unlabeled protein corona, and NPs with a Cy5-labeled protein corona were used for the uptake experiment. The cells were incubated with the NPs for 30 min or 2 h. Subsequently, the NP-containing supernatant was removed after 2 h and replaced with fresh culture medium without NPs. **a.** Flow cytometry was performed to measure the percentual amount of BODIPY (PS NPs) fluorescent cells. Values are shown as the percentage of measured events in regards to the fluorescence. **b.** Percentage of Cy5-positive cells (labeled protein corona) are shown (data is shown as mean ± SD, n = 3). The gating strategy for flow cytometry is provided in Supplementary Figure 2.

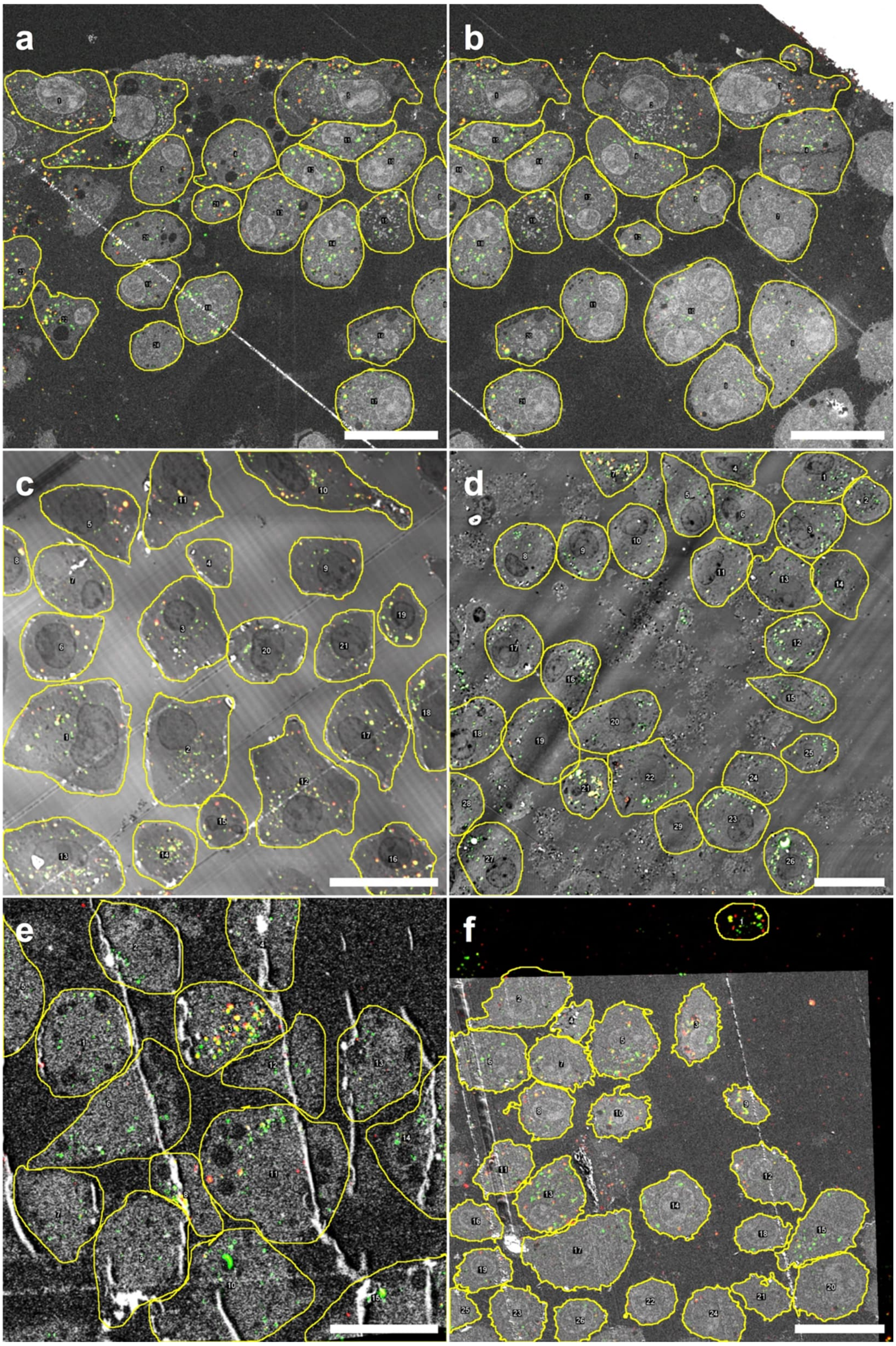

Supplementary Figure 5: Exemplary images of cell selection in connectivity measurements. Identification of cells for a cell-based evaluation of the connectivity of BODIPY and Cy5 signals. **a/b.** Cell selection at 30 min. **c.** Cell selection at 2 h. **d.** Cell selection at 2 h + 4 h. **e.** Cell selection at 2 h + 16 h. **f.** Cell selection at 2 h + 24 h. Scale bars: 20 µm.

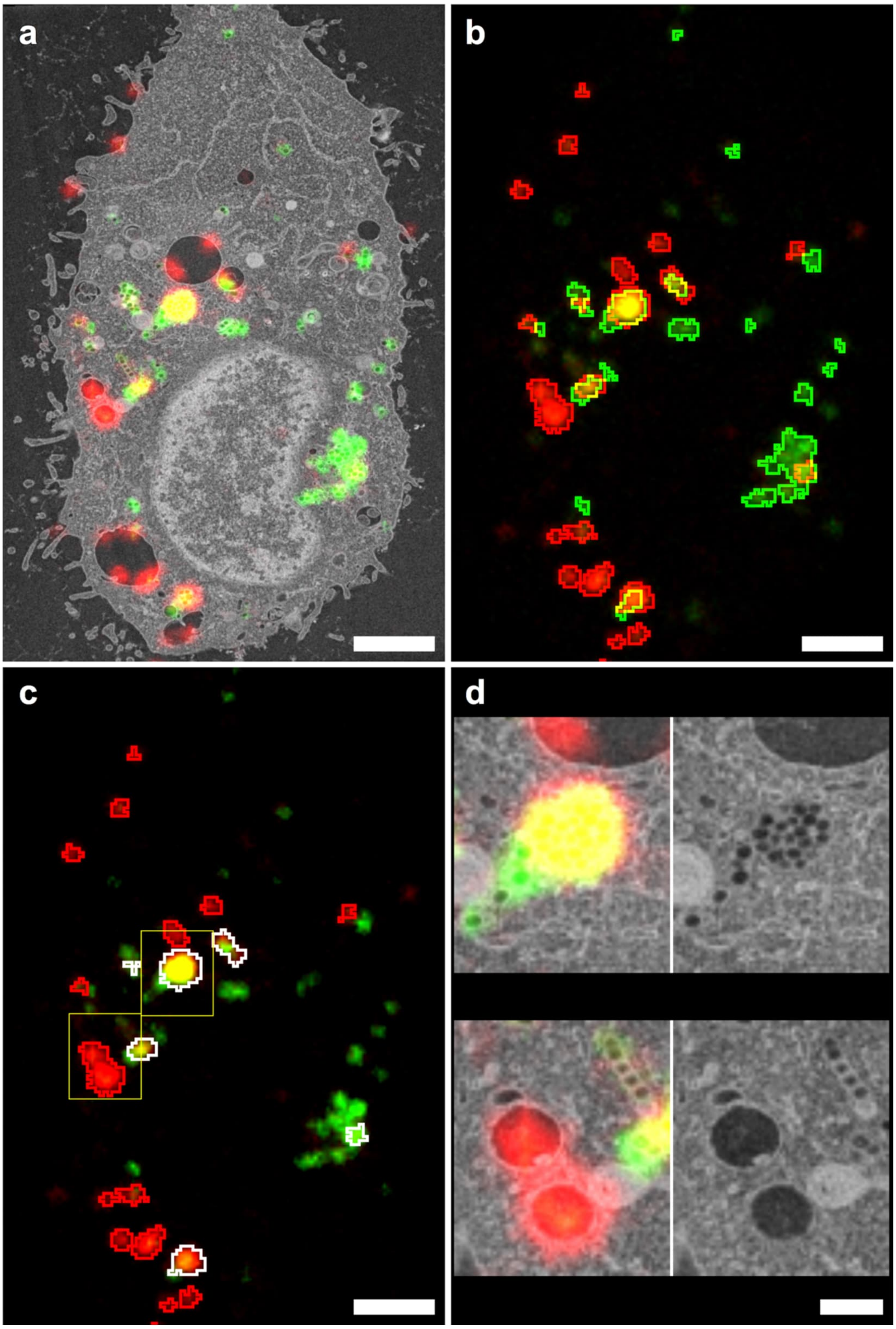

Supplementary Figure 6: Workflow for connectivity measurements. a. Cell to be evaluated. **b.** Thresholding to identify protein and nanoparticle objects. **c.** Classification as connected (white) and not connected (red) protein signal. **d.** Insets of c, showing high-resolution EM images of incomplete (upper) and complete (lower) separation of protein corona from PS NPs Red represents the Cy5-labeled protein corona, green represents BODIPY-labeled PS NPs, yellow represents the overlay of the protein corona and PS NPs. Scale bars a-c: 2 µm. Scale bar d: 500 nm.

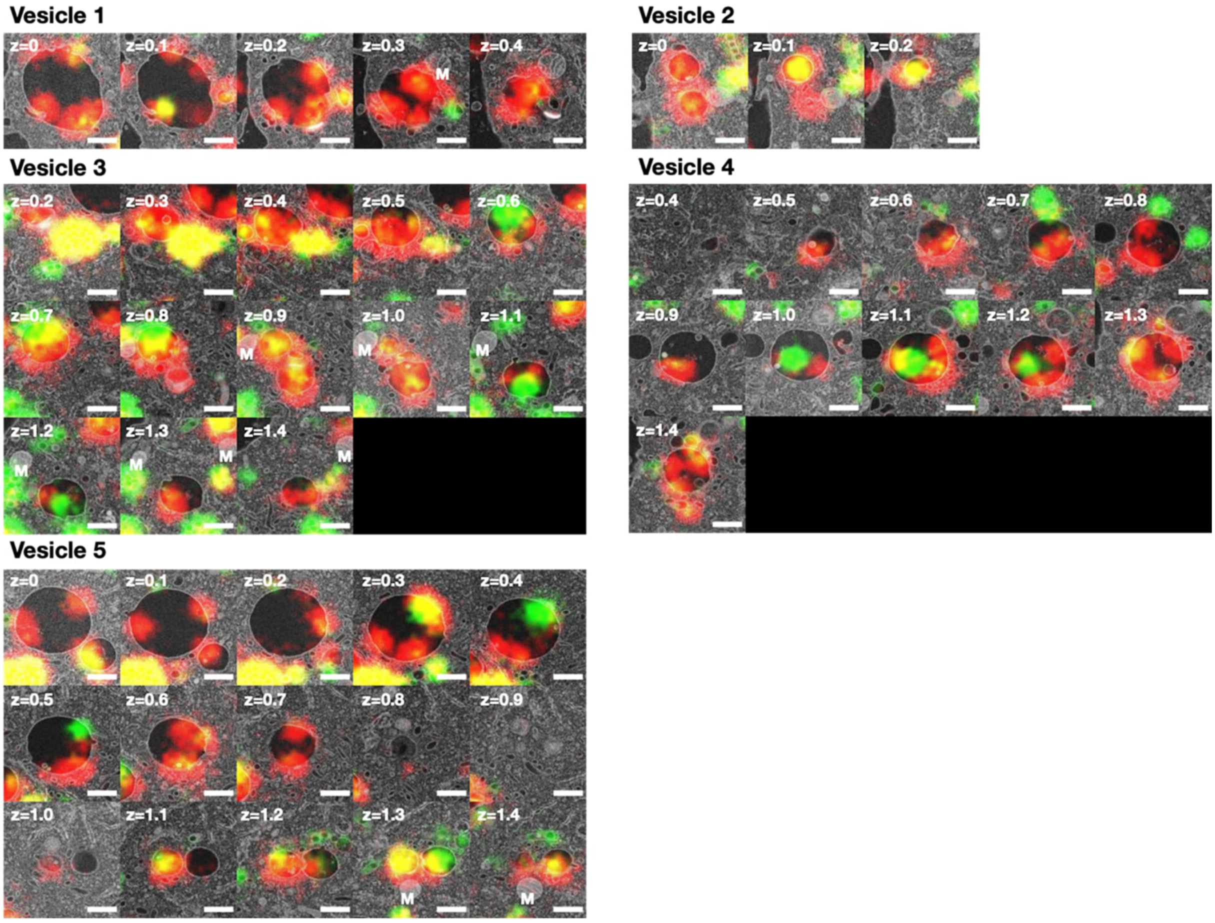

Supplementary Figure 7: Multivesicular bodies (MVBs) containing Cy5-labeled protein corona signal. PS NPs with Cy5-labeled protein corona were incubated with RAW264.7 cells and imaged in CLEM after 2 h + 24 h. z represents the relative depth of the section. M represents mitochondria Red represents the Cy5-labeled protein corona, green represents BODIPY-labeled PS NPs, yellow represents the overlay of the protein corona and PS NPs. Scale bars: 500 nm.

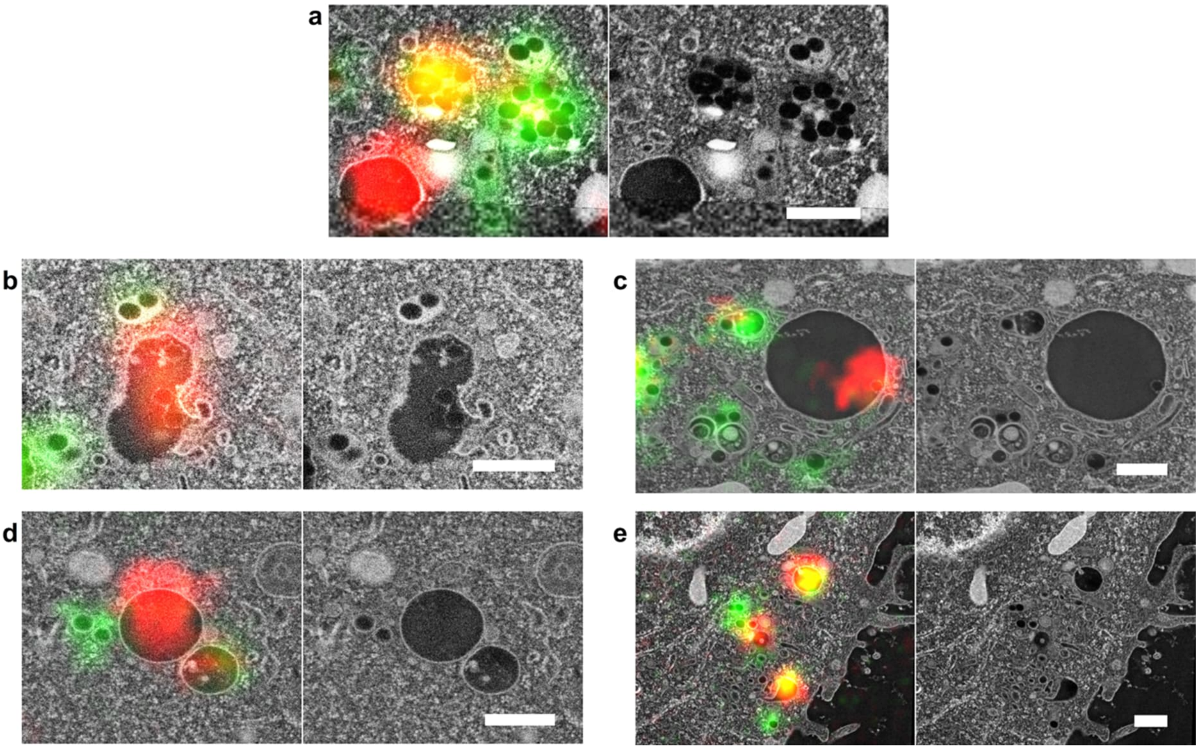

Supplementary Figure 8: Events of separated PS NPs and protein corona in different cells. PS NPs with Cy5-labeled protein corona were incubated with RAW264.7 cells and imaged in CLEM after 2 h + 24 h. **a-e.** Events within five different cells, showing the protein corona-coated PS NPs in crowded round-shaped endosomes, the separated protein corona in MVBs, and the separated PS NPs in tubular recycling endosomes (REs). Red represents the Cy5-labeled protein corona, green represents BODIPY-labeled PS NPs, yellow represents the overlay of the protein corona and PS NPs. Scale bars: 500 nm.

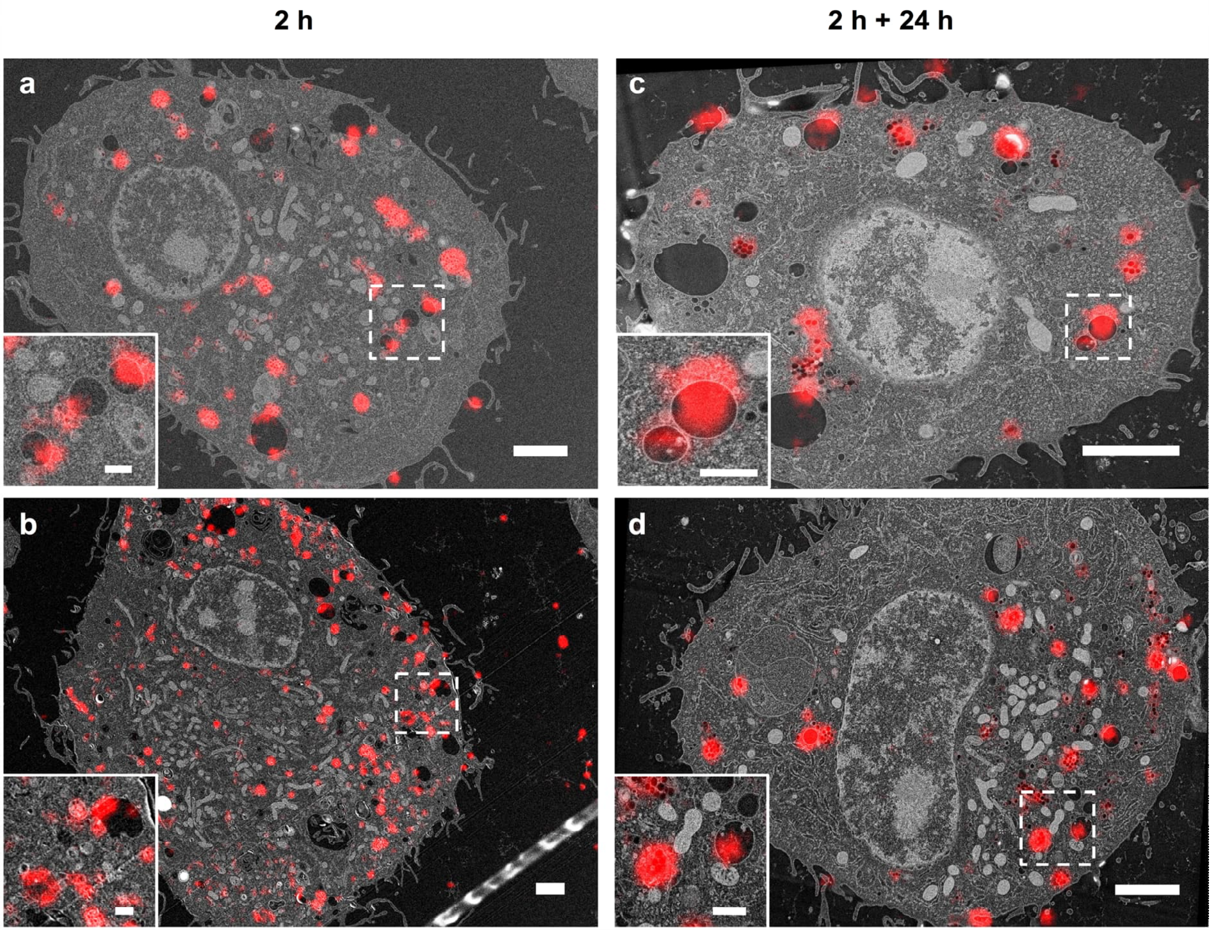

Supplementary Figure 9: Cy5-labeled protein corona at two different time points. PS NPs with Cy5-labeled protein corona were incubated with RAW264.7 cells and imaged in CLEM. **a/b.** Cy5-labeled protein corona located in vesicles after 2 h. **c/d.** Cy5-labeled protein corona located in vesicles after 2 h + 24 h. The sections in the dotted squares were enlarged to highlight the presence of the Cy5 signal within vesicles. Red represents the Cy5-labeled protein corona. The green signal is not included for a better overview of the red signal. Scale bars: 2 µm, Scale bars insets: 500 nm.

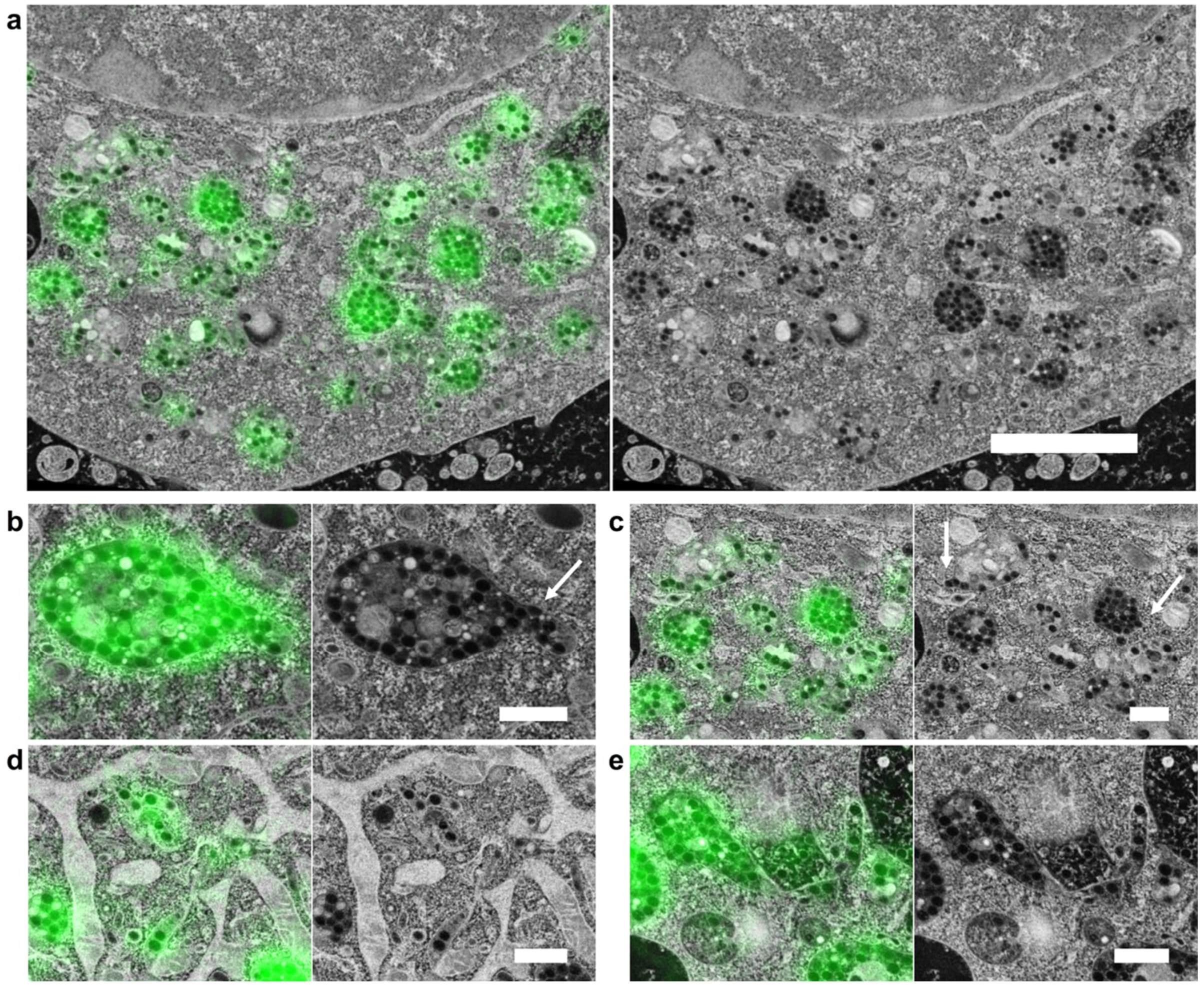

Supplementary Figure 10: Control uptake experiment with PS NPs without protein corona. PS NPs without protein corona were incubated with RAW264.7 cells and imaged in CLEM after 2 h + 24 h. **a.** PS NPs in endosomal vesicles. **b/c.** Vesicles with PS NPs undergo budding and sorting. The white arrows indicate the budding within the vesicles. **d/e.** PS NPs in tubular-shaped REs. Green represents BODIPY-labeled PS NPs. Scale bar a: 2 µm. Scale bars b-e: 500 nm.

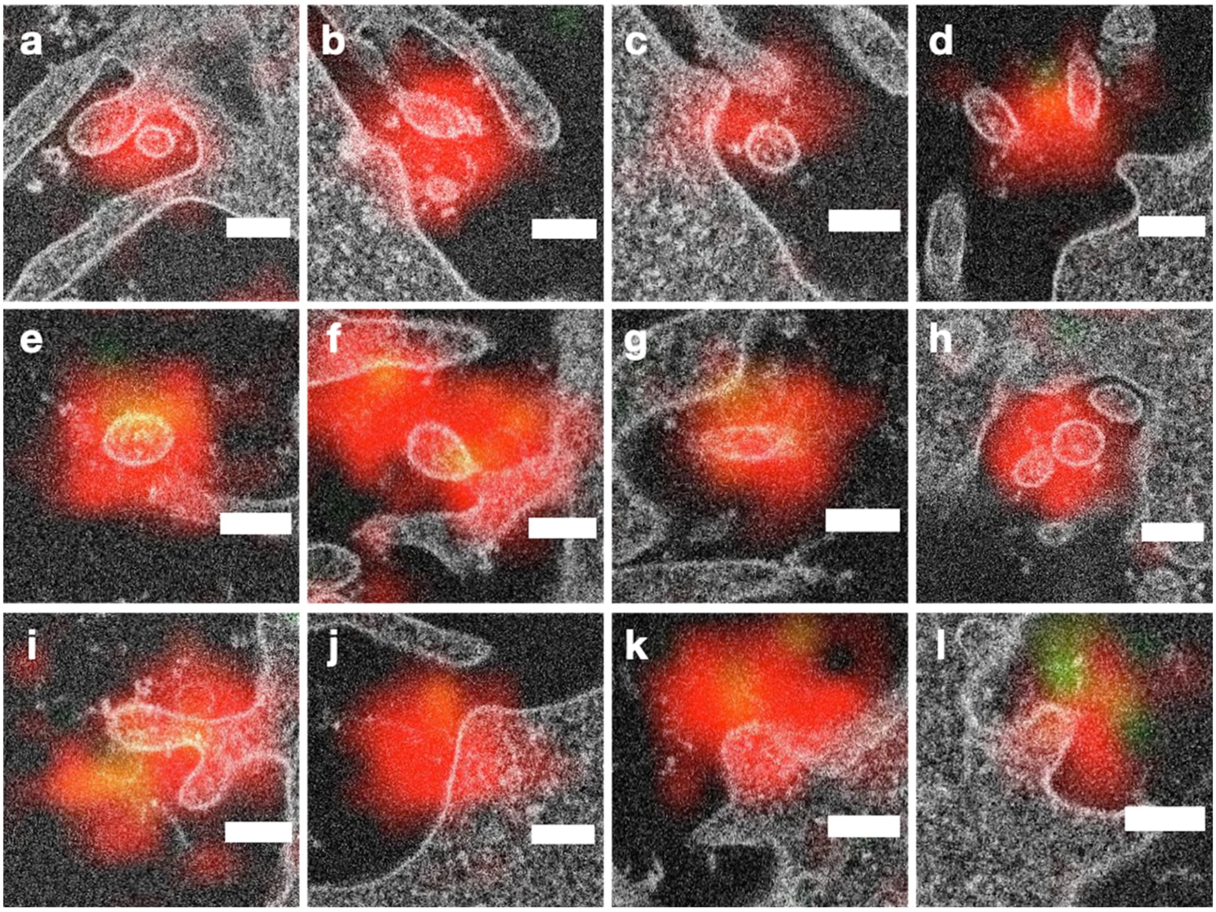

Supplementary Figure 11: CLEM micrographs of extracellular Cy5-labeled protein corona close to the plasma membrane. PS NPs with Cy5-labeled protein corona were incubated with RAW264.7 cells and imaged in CLEM after 2 h + 24 h. **a-h.** Cy5-labeled protein corona located within extracellular vesicles. **i-l.** Cy5-labeled protein corona located next to the plasma membrane. Red represents the Cy5-labeled protein corona. Scale bars: 200 nm.

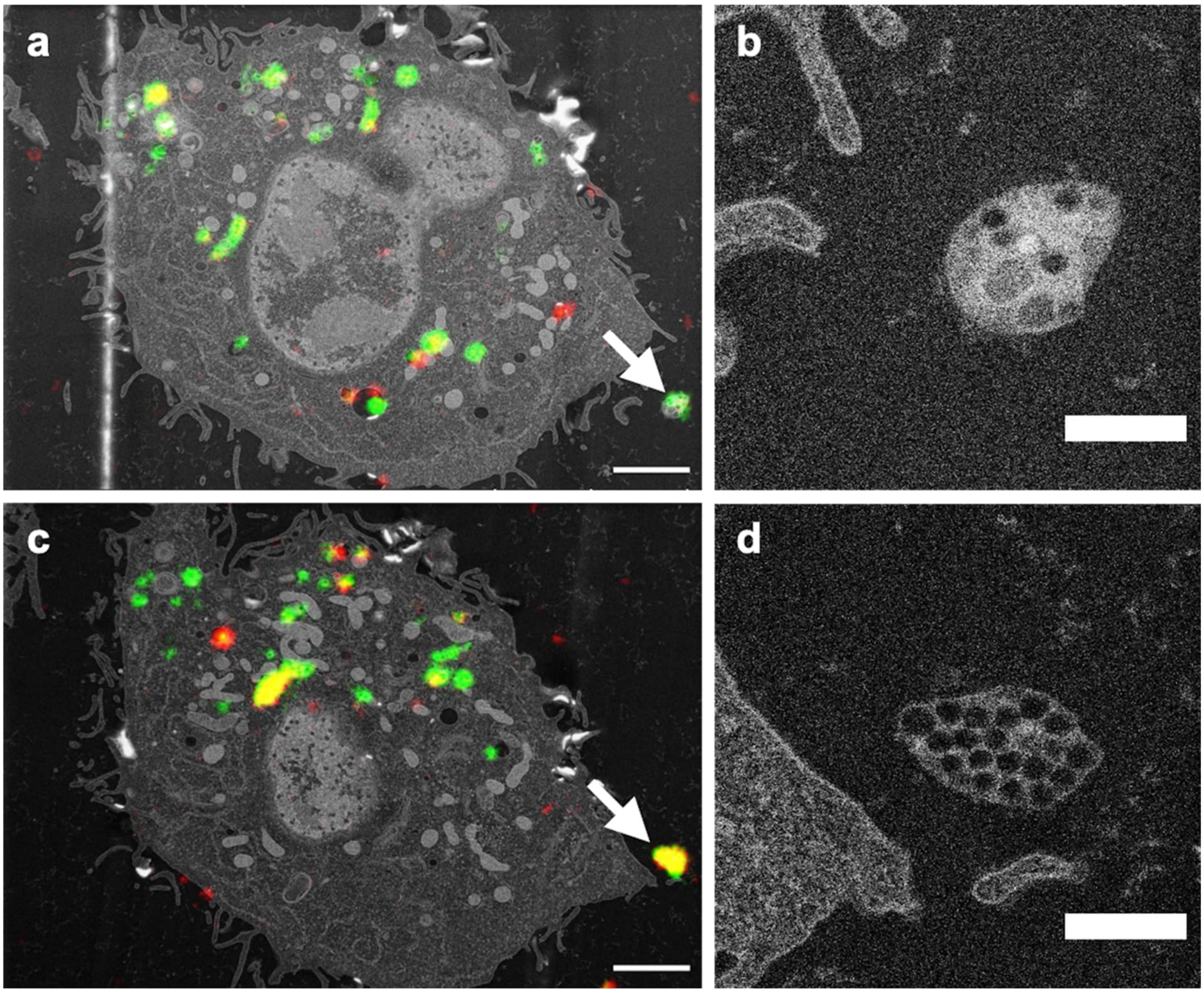

Supplementary Figure 12: Exocytotic vesicles with PS NPs. PS NPs with Cy5-labeled protein corona were incubated with RAW264.7 cells and imaged in CLEM after 2 h + 24 h. **a.** A RAW264.7 macrophage after 2 h + 24 h, arrow indicated the extracellular vesicle with PS NPs nanoparticles. **b.** Zoom-in image of the vesicle in a. **c.** A second macrophage, arrow indicated the extracellular vesicle with protein corona-covered PS NPs. **d.** Zoom-in image of the vesicle in c. Red represents the Cy5-labeled protein corona, green represents BODIPY-labeled PS NPs, yellow represents the overlay of the protein corona and PS NPs. Scale bars: a and c: 2 µm, b and d: 500 nm.

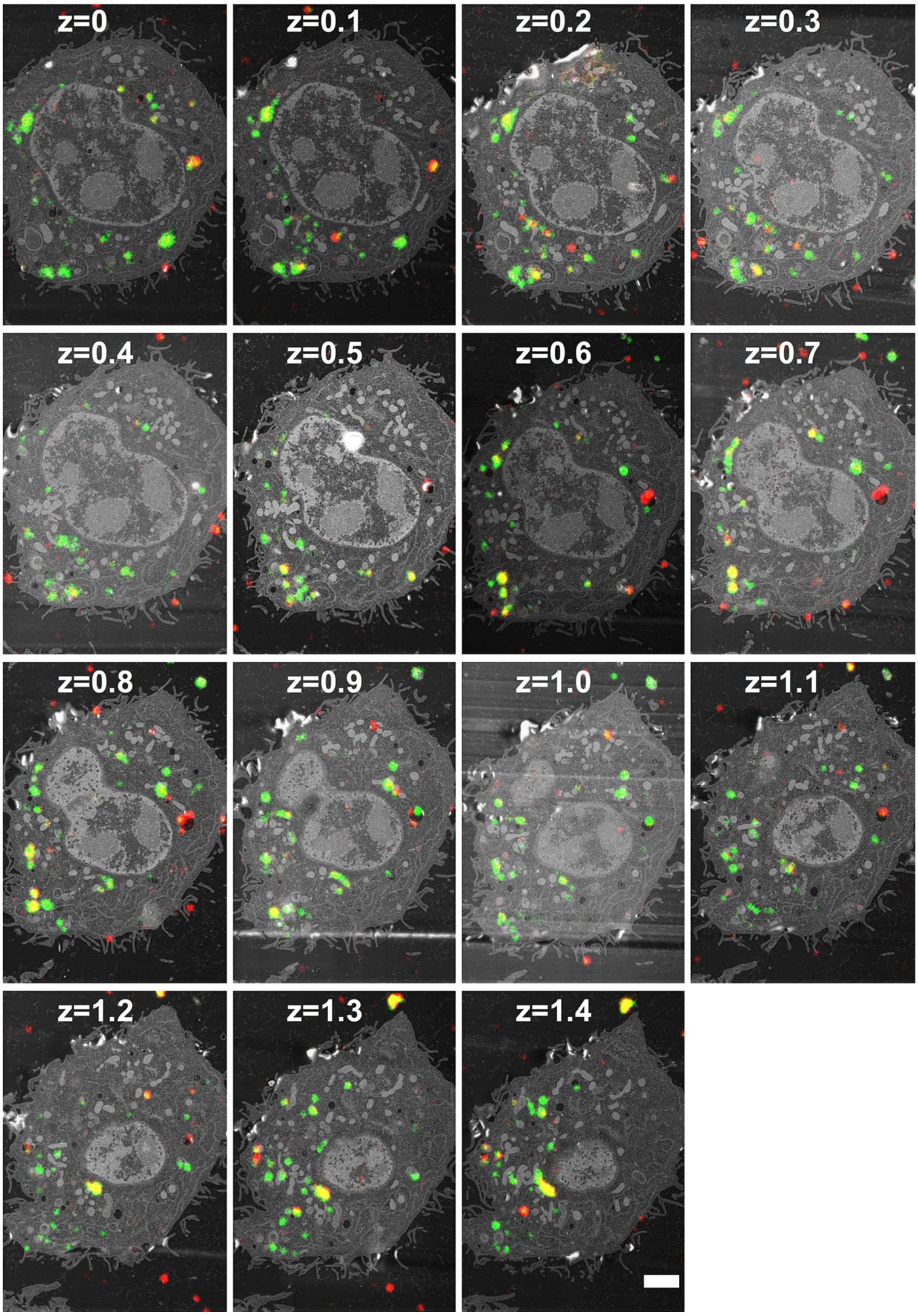

Supplementary Figure 13: Volume CLEM of another cell. PS NPs with Cy5-labeled protein corona were incubated with RAW264.7 cells and imaged in CLEM after 2 h + 24 h. A second cell was inspected with volume CLEM. Supplementary Figure 12a is from z=0.9, Supplementary Figure 12c is from z=1.4. z represents the relative depth of the sections. Red represents the Cy5-labeled protein corona, green represents BODIPY-labeled PS NPs, yellow represents the overlay of the protein corona and PS NPs. Scale bars: 2 µm.

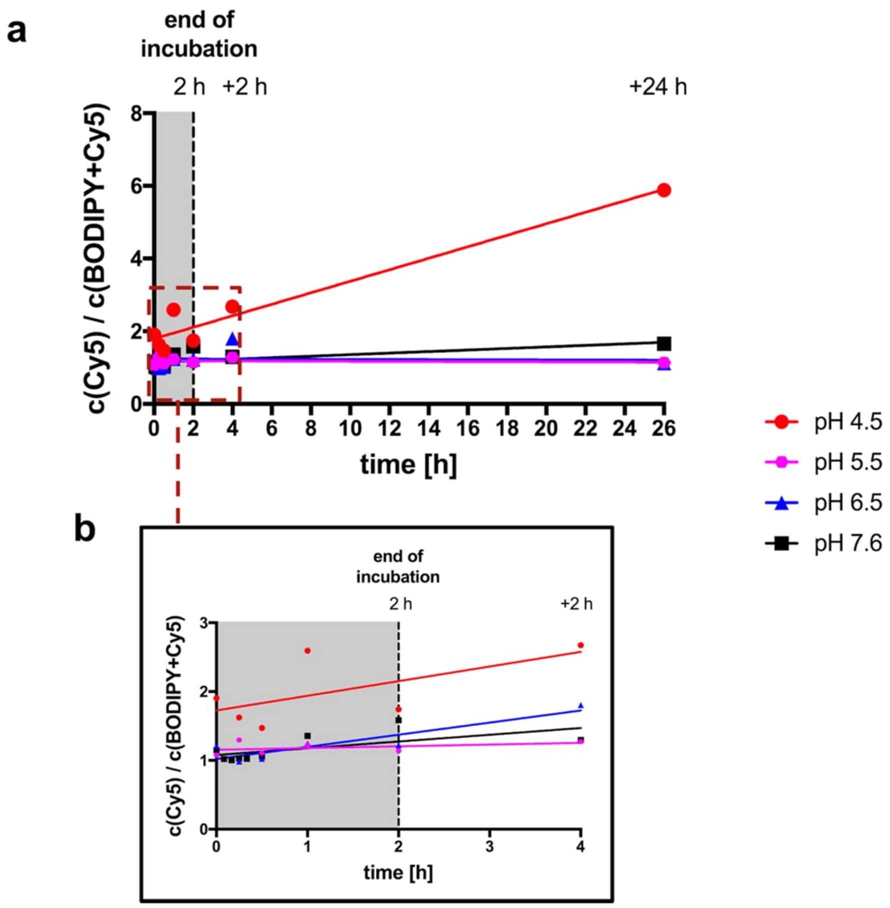

Supplementary Figure 14: Acidification contributes to the separation of the protein corona from PS NPs. PS NPs (BODIPY labeled) with a murine plasma protein corona (Cy5 labeled) were resuspended in cell culture medium with different pH values. The signal correlation was measured by FCCS at various time points **a.** Ratio of the concentrations of Cy5 labeled species (all plasma proteins) to the concentration of the double labeled species (PS NPs with protein corona) vs. incubation time in DMEM at the respective pH. An increase of this ratio indicates partial separation of the protein corona from the nanoparticles. **b.** The data within the dotted square in a. were enlarged to show the measurements in the time frame from 0 h to 4 h. Linear regression was fitted with the data to visualize the correlation progression over time.
